# Characterisation of 20S Proteasome in *Tritrichomonas foetus* and Its Role during the Cell Cycle and Transformation into Endoflagellar Form

**DOI:** 10.1371/journal.pone.0129165

**Published:** 2015-06-05

**Authors:** Antonio Pereira-Neves, Luiz Gonzaga, Rubem F. S. Menna-Barreto, Marlene Benchimol

**Affiliations:** 1 Programa de Pós-graduação em Ciências Morfológicas, Instituto de Ciências Biomédicas, Universidade Federal do Rio de Janeiro, Rio de Janeiro, RJ, Brazil; 2 Instituto Nacional de Ciência e Tecnologia de Biologia Estrutural e Bioimagem, Universidade Federal do Rio de Janeiro, Rio de Janeiro, RJ, Brazil; 3 Fiocruz, Centro de Pesquisa Aggeu Magalhães, Departamento de Microbiologia, Laboratório de Microbiologia e Biologia Celular, Recife, PE, Brazil; 4 Laboratório Nacional de Computação Cientifica (LNCC/MCT), Petrópolis, RJ, Brazil; 5 Fiocruz, Instituto Oswaldo Cruz, Rio de Janeiro, RJ, Brazil; 6 UNIGRANRIO- Universidade do Grande Rio, Duque de Caxias, RJ, Brazil; 7 Instituto de Biofísica Carlos Chagas Filho, Universidade Federal do Rio de Janeiro, Rio de Janeiro, RJ, Brazil; Universidad Autónoma de Madrid, SPAIN

## Abstract

Proteasomes are intracellular complexes that control selective protein degradation in organisms ranging from Archaea to higher eukaryotes. These structures have multiple proteolytic activities that are required for cell differentiation, replication and maintaining cellular homeostasis. Here, we document the presence of the 20S proteasome in the protist parasite *Tritrichomonas foetus*. Complementary techniques, such as a combination of whole genome sequencing technologies, bioinformatics algorithms, cell fractionation and biochemistry and microscopy approaches were used to characterise the 20S proteasome of *T*. *foetus*. The 14 homologues of the typical eukaryotic proteasome subunits were identified in the *T*. *foetus* genome. Alignment analyses showed that the main regulatory and catalytic domains of the proteasome were conserved in the predicted amino acid sequences from *T*. *foetus*-proteasome subunits. Immunofluorescence assays using an anti-proteasome antibody revealed a labelling distributed throughout the cytosol as punctate cytoplasmic structures and in the perinuclear region. Electron microscopy of a *T*. *foetus*-proteasome-enriched fraction confirmed the presence of particles that resembled the typical eukaryotic 20S proteasome. Fluorogenic assays using specific peptidyl substrates detected presence of the three typical peptidase activities of eukaryotic proteasomes in *T*. *foetus*. As expected, these peptidase activities were inhibited by lactacystin, a well-known specific proteasome inhibitor, and were not affected by inhibitors of serine or cysteine proteases. During the transformation of *T*. *foetus* to endoflagellar form (EFF), also known as pseudocyst, we observed correlations between the EFF formation rates, increases in the proteasome activities and reduced levels of ubiquitin-protein conjugates. The growth, cell cycle and EFF transformation of *T*. *foetus* were inhibited after treatment with lactacystin in a dose-dependent manner. Lactacystin treatment also resulted in an accumulation of ubiquitinated proteins and caused increase in the amount of endoplasmic reticulum membranes in the parasite. Taken together, our results suggest that the ubiquitin-proteasome pathway is required for cell cycle and EFF transformation in *T*. *foetus*.

## Introduction

The protist *Tritrichomonas foetus* (Excavata, Parabasalia) is an important pathogen that causes bovine and feline trichomonosis. Bovine trichomonosis is a venereal disease that leads to reproductive failure in infected herds, resulting in considerable economic burden in beef-producing areas where open range management and natural breeding are practiced [1]. Feline trichomonosis is a large-bowel disease that affects domestic cats worldwide [2]. In addition to its economic and veterinary importance, *T*. *foetus* is also of interest from the perspective of cell biology. Similar to the related human pathogen *Trichomonas vaginalis*, *T*. *foetus* contains cell structures commonly found in eukaryotes, e.g. endoplasmic reticulum (ER) and Golgi complex. However, it also contains unusual anaerobic energy-generating organelles called hydrogenosomes and a very peculiar cytoskeleton that includes a microtubular pelta-axostylar system, the costa, a large striated root, among others [3]. Like other parabasalids, *T*. *foetus* has a crucial position in various schemes of eukaryotic evolution and presents a large genome, which makes it a fascinating model for evolutionary studies [4].

*T*. *foetus* has a simple life cycle that consists of only a trophozoitic form, which is characterised by a pear-shaped (PS) body, three anterior flagella and one recurrent flagellum. However, under stress, such as low temperature or the presence of drugs, e.g. colchicine, the trophozoite takes on an endoflagellar form (EFF), also known as pseudocyst. In this form, the parasite adopts a spherical or ellipsoid shape and internalises its flagella, but no cyst wall surrounds the cell [5]. The EFF is a reversible form commonly found in preputial secretions from *T*. *foetus*-infected bulls [6] and exhibits a distinct mitotic mechanism, called budding, when compared to PS parasites [7]. In addition to morphological and behavioral alterations, some genes are differentially expressed in the trichomonads’ EFFs [8–10], suggesting that proteins are selectively synthesised and degraded during the transformation from PS to EFF.

Selective protein degradation plays an important role in events that are critical to the control of many biological processes [11]. In this context, the ubiquitin-proteasome system is the major proteolytic pathway responsible for selective turnover and breakdown of damaged, misfolded and short-lived proteins in the cytosol and nucleus of eukaryotic cells [12]. Proteins degraded by this pathway are covalently conjugated to multiple ubiquitin (Ub) molecules (poly-Ub) and then they are recognised, unfolded and broken down into smaller peptides by the 26S proteasome, a 2,000-kDa ATP-dependent proteolytic complex [12]. Proteasomal proteolysis is crucial for the maintenance of cellular homeostasis because it prevents the toxic accumulation of abnormal proteins and regulates a wide range of cellular processes, such as protein quality control, cell cycle progression, cell differentiation, gene transcription control, DNA repair, cell death and antigen processing [12]. The catalytic core of the 26S proteasome is a 700-kDa multi-subunit threonine protease known as 20S proteasome. This core particle is found in organisms from the three major domains of life, Eubacteria, Archaea and Eukarya. In eukaryotes, the 20S proteasome is a barrel-shaped structure that consists of four stacked heptameric rings in a α_1–7_β_1–7_β_1–7_α_1–7_ organisation, forming a central cavity [12–14].

In some parasitc protists, the 20S proteasome has already been identified, such as in *Trypanosoma* spp., *Leishmania* spp., *Entamoeba* spp., *Plasmodium* spp., *Toxoplasma gondii* and *Giardia lamblia* [14]. In these organisms, proteasomal proteolysis is required for replication, life stage-specific transformation and metabolic adaptation to environment changes or stress responses and could therefore be a promising therapeutic target [11, 13–14]. There is genetic evidence that the Ub-proteasome system is present in *T*. *vaginalis* [13, 15]. Although an Ub gene has been found in *T*. *foetus* [16], the 20S proteasome has not yet been identified in this parasite. In addition, the biochemical properties and biological functions of the proteasomes in trichomonads remain unknown. Consequently, in this study, we used complementary techniques, such as a combination of *de novo* whole genome sequencing technologies, bioinformatic algorithms, cell fractionation, and biochemistry and microscopy approaches, to identify and characterise the 20S proteasome of *T*. *foetus*. The participation of proteasomal proteolysis in the *T*. *foetus* cell cycle and during the process of transformation in EFF was also investigated.

## Results and Discussion

### Conditions for experimental assays

PS parasites, those that exhibit a pear-shaped body with at least one visible external flagellum (S1A Fig), from axenic cultures maintained under standard conditions and EFF under a temperature-based assay were taken [5, 7]. The EFFs are those rounded or ellipsoid parasites that have no visible external flagella (S1B Fig). Only populations that contained greater than 90% of parasites in either PS or EFF were used (S1 Fig). The viability of the PS and EFF in each sample remained unaltered (not shown).

### Identification and characterisation of the predicted *T*. *foetus*-20S proteasomal sequences

Because there are no reports concerning proteasomes in *T*. *foetus*, and a fully-sequenced parasite genome is not yet available, we initially applied a combination of both Roche-454 (shotgun library) [17] and Illumina (mate pair library) [18] sequencing technologies to generate sequence reads with predicted open reading frames (ORFs) for the 20S proteasomal subunits-genes in *T*. *foetus*. The combined application of these two tools produces higher-quality assemblies and better results than those using any sequencing technology alone [19–21].

In contrast to archaeal proteasomes, which are composed of seven identical α-type and β-type subunits, the 20S proteasomes from eukaryotes are characterised by seven different α-type and at least seven different β-type subunits clustered by sequence similarity into 14 distinct groups: α1 to α7 and β1 to β7 [22]. The α-type subunits have no enzyme activity and play structural roles, such as assisting in proteasome assembly, serving as selective barriers of substrates to the central proteolytic cavity and as docking domains for several regulatory particles [13–14, 22]. The proteolytic sites are located on the β-type subunits, which carry the catalytic N-terminal threonine residues facing the inner surface of the central cavity enclosed by the two β-rings [12, 14]. Here, we identified the homologues of these 14 proteasomal subunits in the *T*. *foetus* genome (Table 1). For this purpose, Illumina reads were aligned to 454 contigs to produce a 454/Illumina consensus sequence. Then, 14 sequences of the *T*. *vaginalis*-20S proteasome subunit genes were used as references to guide the assembly of the proteasome genes in *T*. *foetus* (Table 1).

**Table 1:** Summary of the predicted 20S-proteasome proteins identified in *T*. *foetus* shotgun (454) and mate pair (Illumina) libraries using selected protein sequences of the *T*. *vaginalis* as reference.

20S proteasomal proteins identified in *T*. *foetus*							Reference *T*. *vaginalis* protein sequences		
Acession number ^a^	Product orthology^b^	Name	Length (aa)^c^	Score	E-value	Number of gene copies	KO-Entry	NCBI-RefSeq	Length (aa)^c^
KF428747	20S proteasome subunit alpha 1	TfoetusA1	241	323	4.00E-86	2	K02730	XP_001312891.1	241
KF428748	20S proteasome subunit alpha 2	TfoetusA2	231	390	1.00E-106	1	K02726	XP_001307739.1	232
KF428749	20S proteasome subunit alpha 3	TfoetusA3	251	462	1.00E-128	2	K02728	XP_001304820.1	251
KF428750	20S proteasome subunit alpha 4	TfoetusA4	235	383	1.00E-104	1	K02731	XP_001328403.1	235
KF428751	20S proteasome subunit alpha 5	TfoetusA5	250	427	1.00E-117	2	K02729	XP_001310279.1	251
KF428752	20S proteasome subunit alpha 6	TfoetusA6	235	358	1.00E-96	2	K02725	XP_001325659.1	233
KF428753	20S proteasome subunit alpha 7	TfoetusA7	233	318	8.00E-85	1	K02727	XP_001584200.1	240
KF428754	20S proteasome subunit beta 1	TfoetusB1	217	214	1.00E-053	2	K02738	XP_001323441.1	216
KF428755	20S proteasome subunit beta 2	TfoetusB2	264	329	4.00E-088	1	K02739	XP_001313725.1	275
KF428756	20S proteasome subunit beta 3	TfoetusB3	207	314	1.00E-083	2	K02735	XP_001313488.1	206
KF428757	20S proteasome subunit beta 4	TfoetusB4	191	306	3.00E-081	1	K02734	XP_001311576.1	191
KF428758	20S proteasome subunit beta 5	TfoetusB5	258	398	1.00E-108	1	K02737	XP_001582522.1	256
KF428759	20S proteasome subunit beta 6	TfoetusB6	228	287	6.00E-095	1	K02732	XP_001312251.1	224
KF428760	20S proteasome subunit beta 7	TfoetusB7	225	229	5.00E-058	1	K02736	XP_001313319.1	214

^a^ The sequences were deposited in GenBank

^b^ Assigned according to the KEGG Orthology database

^c^ aa, number of amino acids

**Fig 1:**
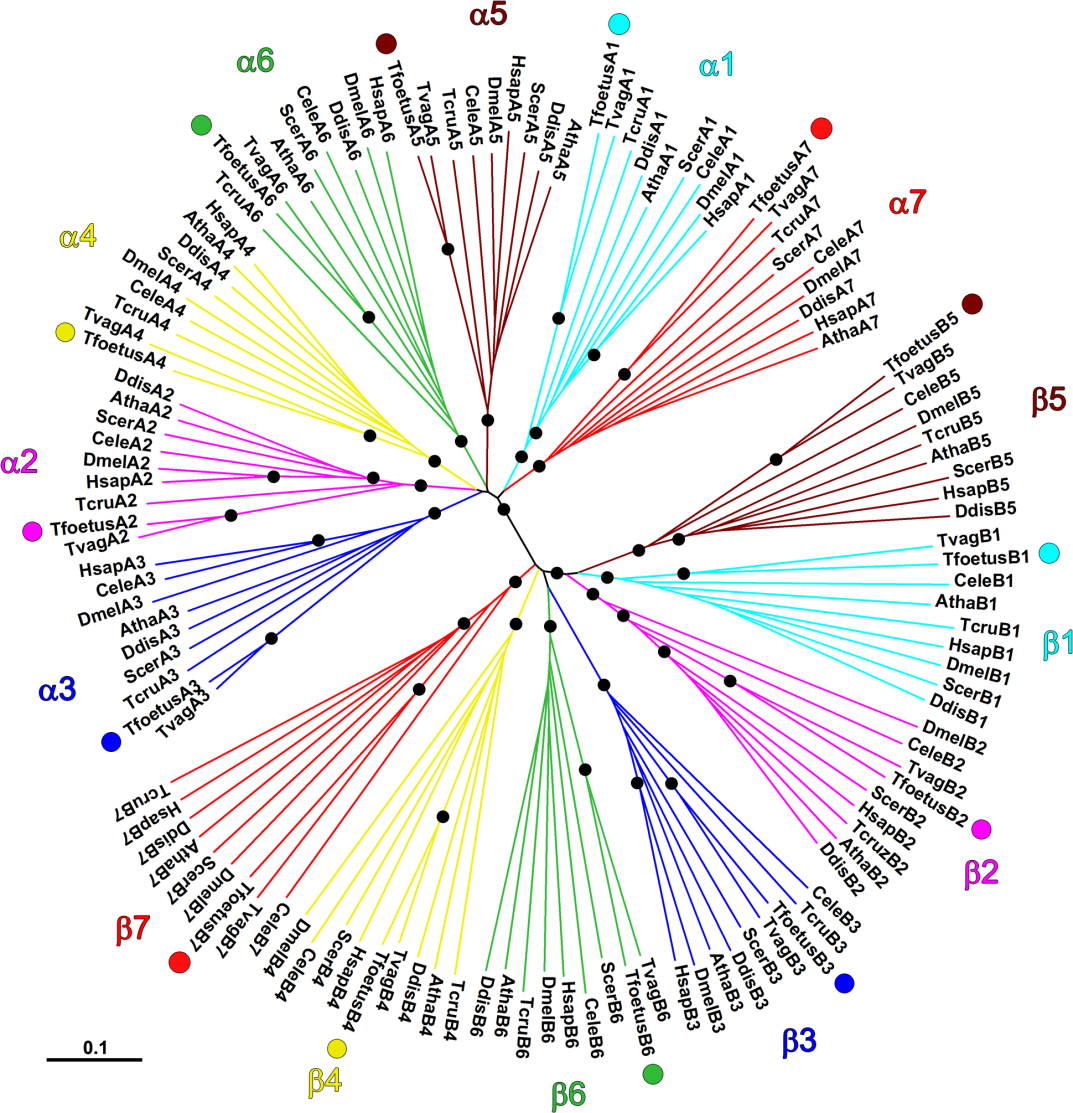
Phylogenetic analysis of the predicted 20S proteasome α- and β-type subunits from *T*. *foetus*. The amino acid sequences of the 14 proteasome subunits identified in the *T*. *foetus* genome were aligned with their respective orthologues sampled from several eukaryotic species using CLUSTAL W algorithm. The unrooted phylogenetic tree was constructed by the neighbor-joining method based on the alignment using MEGA v. 5.2.2 software. The distance matrix was obtained by calculating p-distances for all pairs of sequences. Gaps were excluded using the pairwise-deletion option. Branch points were tested for significance by bootstrapping using 1,000 replications. All seven α- and β-type subunits are marked and shown in different colours. The *T*. *foetus* 20S proteasome subunits are indicated with colourful dots. Nodes supported by high bootstrap results (≥ 95%) are indicated by black dots. Scale bar represents 0.1 substitutions per site. Organism abbreviations: Tvag, *Trichomonas vaginalis*; Tcru, *Trypanosoma cruzi*; Scer, *Saccharomyces cerevisiae*; Ddis, *Dictyostelium discoideum*; Cele, *Caenorhabditis elegans*; Atha, *Arabidopsis thaliana*; Dmel, *Drosophila melanogaster*; Hsap, *Homo sapiens*. See Table 1 and S1 Table for the accession numbers of the proteasome sequences.

The proteasome genes found were used to derive the predicted full-length amino acid sequences of the corresponding proteins (Table 1; S2 and S3 Figs). *In silico* analysis using motif-finding algorithms identified specific well-conserved proteasome α-type subunit domains in seven *T*. *foetus* amino acids sequences (S2 Fig) and proteasomal β-type subunit motifs in the other seven predicted sequences (S3 Fig). To further assign the paralogy and orthology between the sequences of *T*. *foetus*-20S proteasome subunits, unrooted phylogenetic analyses were performed using selected proteasome sequences from a wide taxonomic range of species (Fig 1). S1 Table provides the accession numbers and names of the proteasome sequences assigned for each species used in the phylogenetic analyses. The sequences of the *T*. *foetus* 20S proteasome identified here were clustered into 14 distinct monophyletic subgroups corresponding to the previously-defined α- and β-type subunits (Fig 1). These results are supported by high bootstrapping values (≥950/1,000; Fig 1). Therefore, taken together, our data indicate that the subunits of the *T*. *foetus* 20S proteasome were assigned to the 14 different groups as a typical proteasome from eukaryotes. The identification of seven α- and β-type proteasome subunit genes in *T*. *foetus*, one of the deepest-branching eukaryotes, suggests that the duplications that gave rise to distinct α- and β-paralogues are ancient events that occurred very early during the evolution of Eukarya. This is consistent with other studies that indicate the presence of multiple proteasome subunits in other species from early-branching eukaryotes, such as *G*. *lamblia*, kinetoplastids and Microsporidia [13–14, 22–23].

In yeast, each of the 20S proteasome subunit genes is found as a single copy [24]. In contrast, here, a single gene was identified for eight of the *T*. *foetus*-proteasomal subunits (α2, α4, α7, β2 and β4–7), whereas duplicated gene copies were detected for each of the remaining six subunits (α1, α3, α5–6, β1 and β3; Table 1). The identities/similarities between the predicted amino acid sequences derived from the related pairs of the duplicated *T*. *foetus*-proteasomal genes varied in a range of 51/71 to 97/98%, with a mean of 83/91% (not shown). These results were expected, because in addition to being an important evolutionary mechanism, the gene duplication of several protein families is a very common event in trichomonads [4, 15]. Similarly, duplicated proteasome subunit genes are described in several species, including *T*. *vaginalis* [15], *Arabidopsis* [25] and *Drosophila* spp. [26]. Although the biological significance of the genetic redundancy of the 20S proteasome subunits is not yet clear, there is evidence that duplications could provide backup copies and/or ensure that sufficient amounts of the corresponding subunits are produced [25–26]. Alternatively, pairs of paralogues might be differentially regulated to allow synthesis of the subunits across a wide range of developmental states and environmental conditions [25–26]. Another possibility is that duplicated genes encode proteins that impart distinct proteolytic specificities and/or functions to the 20S proteasome [25–26]. Further studies are necessary to determine the functional significance of the duplicated proteasome genes in *T*. *foetus*.

**Fig 2:**
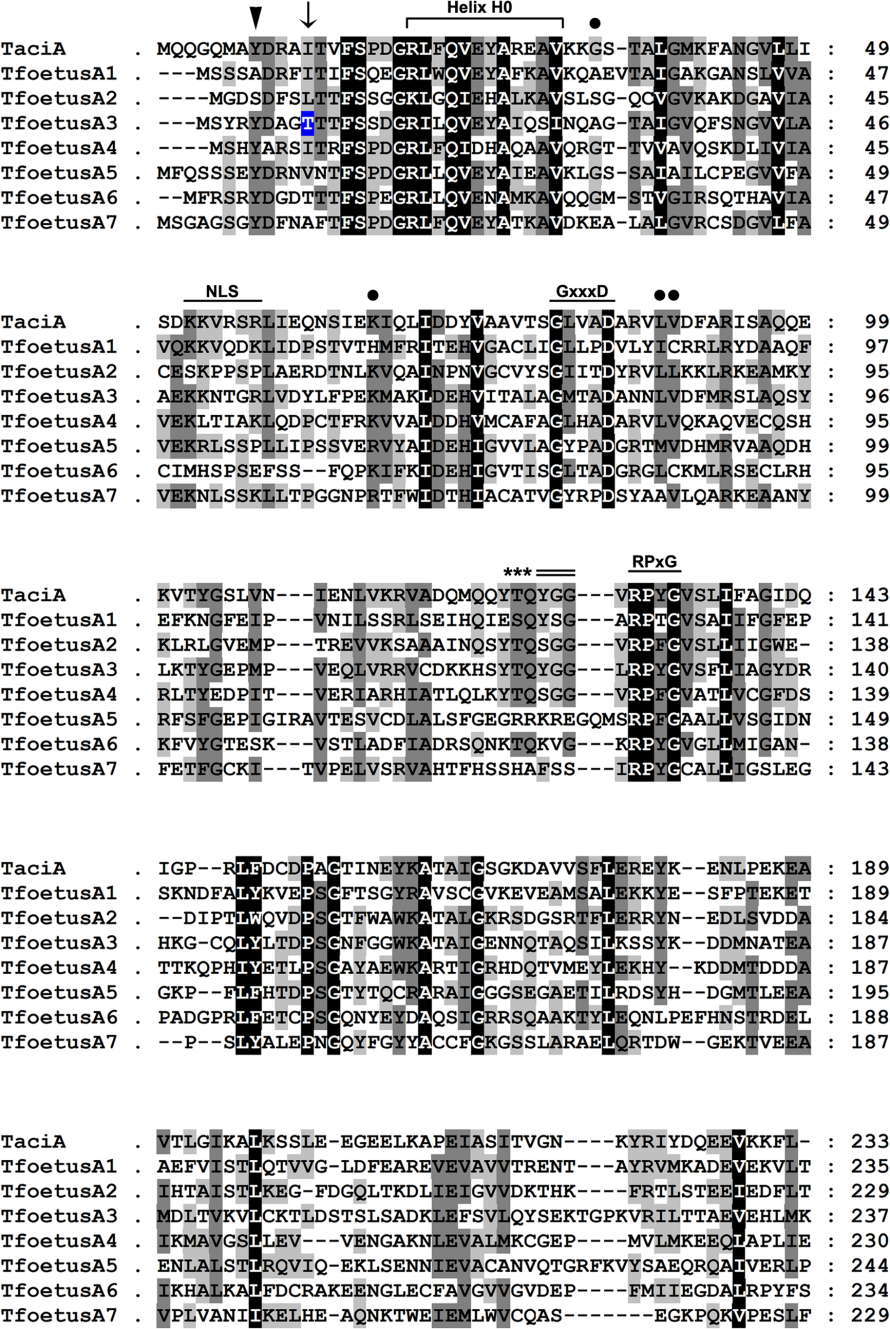
Amino acid sequence alignment of the *T*. *foetus* proteasomal α-type subunits. The alignment was performed using the CLUSTAL W program. The α subunit from the Archaea *Thermoplasma acidophilum* (TaciA—UniProt accession: P25156) was used as a reference to identify the conserved domains in the α-type subunits from *T*. *foetus*. Dashes indicate gaps introduced in the protein sequence alignment. Residues that are functionally conserved in all subunits are shaded in black. Residues that are functionally conserved in 75% to 87.5% and 50% to 62.5% of the subunits are shaded in dark and light grey, respectively. The N-terminal Tyr residue essential for assembly of the α ring is indicated by the arrowhead. The bracket indicates the N-terminal α-helical region (helix H0) responsible for interactions between the α-type subunits and their assembly into rings. The arrow indicates the position of the N-terminal Thr residue in the α3 subunit (shaded in blue) essential to regulate the gate-opening to the central proteolytic cavity of the proteasome. The highly conserved proteasome GxxxD motif (x represents any residue) and the α-family RPxG motif (x represents a hydrophobic residue) responsible for forming the base of the loop that constricts the central pore at the level of the α rings are indicated. The putative nuclear localisation signal sequence K(K/R)xxx(K/R) (x represents any residue) is also indicated. The asterisks show the amino acids at the turn region of the *T*. *acidophilum* α subunit and the double line identifies the amino acids that border the pore of the archaeal α rings. Circles indicate Gly-34, Lys-66, Leu-81 and Val-82 residues essential for binding of the ATPase regulatory particles to the 20S proteasome from *T*. *acidophilum*.

**Fig 3:**
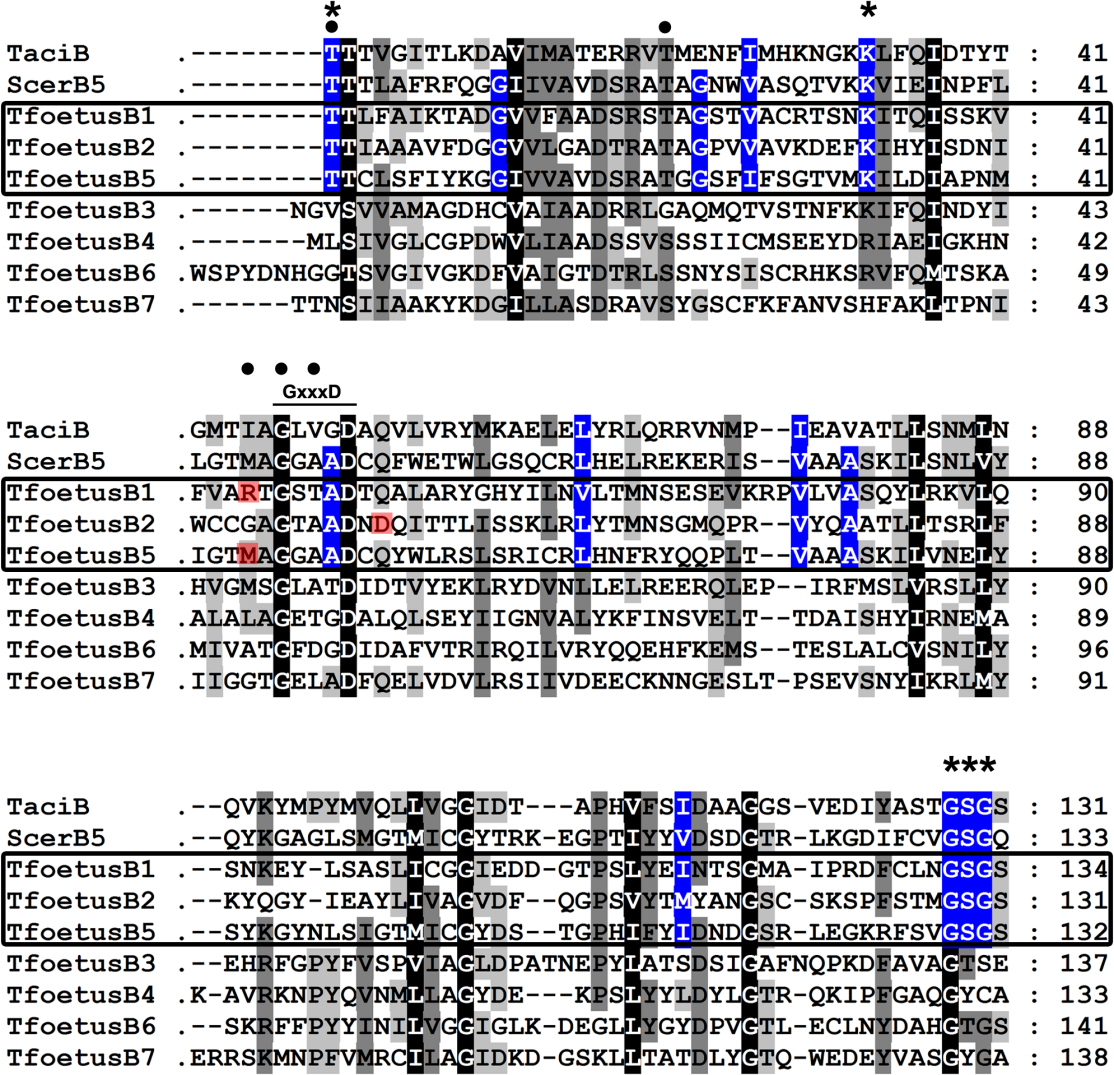
Amino acid sequence alignment of the proteasomal β-type subunits from *T*. *foetus*. The alignment was performed using the CLUSTAL W program. The catalytically active β subunit from the Archaea *T*. *acidophilum* (TaciB—UniProt accession: P28061) and β5-subunit of *S*. *cerevisiae* were used as references to identify the conserved domains in the β-type subunits from *T*. *foetus*. For the purpose of clarity, the alignment was performed using the sequences that were previously identified by the NCBI CD-Search software (see S3 Fig for details). Dashes indicate gaps introduced in the protein sequence alignment. Residues that are functionally conserved in all subunits are shaded in black. Residues that are functionally conserved in 75% to 87.5% and 50% to 62.5% of the subunits are shaded in dark and light grey, respectively. The box indicates the three possible catalytically active subunits of the *T*. *foetus* 20S proteasome. The functionally conserved residues found in the proteolytic active subunits are shaded in blue. Asterisks indicate the N-terminal nucleophile Thr-1 residue as well the catalytically essential Lys-33, Gly-128, Ser-129, and Gly-130 residues in the proteolytic active β-type subunits. The highly-conserved proteasome GxxxD motif (x represents any residue) is indicated. The positions of residues responsible for caspase-like, trypsin-like and chymotrypsin-like activities in the yeast subunits β1, β2 and β5, respectively, are shaded in red. Circles indicate the conserved Thr-1, Thr-21, Met-45, Gly-47 and Ala-49 residues of subunit β5 from yeasts that interact with the inhibitor lactacystin.

In agreement with the data reported for other eukaryotes [22, 25], our BLAST analyses revealed that the *T*. *foetus* α-subunits were generally more related among themselves (S2 Table) than were the β-subunits (S3 Table). In addition, similar to other species [13, 22, 25], the amino acid sequences of each α- and β-subunit from *T*. *foetus* were more similar to their respective related orthologues in several organisms than to the sequences of *T*. *foetus*-paralogous subunits (Figs 2 and 3; S2–S5 Tables). Taken together, these results further support that the α- and β-subunits were derived from a common ancestral gene and that the splitting into seven subtypes in each family probably occurred very early during the divergence of the main eukaryotic kingdoms [13, 22, 25].

### Sequence analysis of potential regulatory motifs in the predicted *T*. *foetus*-α and β subunits

Assuming that the regulatory and catalytic mechanisms of proteasomes are evolutively conserved [22, 25], amino acid sequence alignments were performed using the α- and β-type subunits from the Archaea *Thermoplasma acidophilum* as references to identify conserved domains in the α- and β-type subunits from *T*. *foetus* (Figs 2 and 3).

The most highly-conserved domain found in the α-type subunits of the proteasome from Archaea to Eukarya is the N-terminal α-helical region, known as helix H0, which is responsible for interactions between the α-subunits, their assembly into rings and their interactions with regulatory complexes [25, 27–28]. α-Type subunits also contain two further highly-conserved sequence motifs: an RPxG motif (x represents a hydrophobic residue), responsible for forming the base of the loop that constricts the central pore at the level of the α rings, and a GxxxD motif (x represents any residue), which is also conserved in β-type subunits and may play a role in determining the size and rigidity of the central pore constriction [27, 29–30]. In agreement, the helix H0 region, RPxG and GxxxD motifs were found in all *T*. *foetus* α-type subunits (Fig 2). The GxxxD motif was also observed in all *T*. *foetus* β-type subunits (Fig 3).The highly-conserved N-terminal Tyr residue, which is essential for assembly of the α-ring in archaeal and eukaryotic proteasomes [25, 31], was found in five *T*. *foetus* α-type subunits (α3-α7; Fig 2). The Tyr-126 and Gly-128 residues that border the pore of the archaeal α rings [26,29] were observed in two *T*. *foetus* α-type subunits (α1 and α3; Fig 2). The Tyr-123, Thr-124 and Gln125 residues of the turn region of the *T*. *acidophilum* α-type subunit [29] were found in three *T*. *foetus* α-type subunits (α2-α4; Fig 2). Moreover, a highly-conserved N-terminal Thr residue, located in the α3 subunit from eukaryotes and essential for the regulation of gate-opening to the central proteolytic cavity of the proteasome [15, 22], was also found in the *T*. *foetus* α3 subunit sequence (Fig 2).

The Gly-34, Lys-66, Leu-81 and Val-82 residues, which are essential for the binding of the ATPase regulatory particles to the archaeal 20S proteasome, are conserved in the most of the α-type subunits of the eukaryotic cells [28] and were also observed in almost all *T*. *foetus* α-type subunits (Fig 2). In addition, the subunits α1 and α3 from *T*. *foetus* displayed a putative nuclear localisation signal (NLS) K(K/R)xxx(K/R) sequence (Fig 2), which is found in α-type subunits from *T*. *acidophilum* and eukaryotes [26, 31–32]. The NLS sequence is not conserved between different α-type subunits of the eukaryotic cells and this motif is responsible for regulating the nuclear/cytosol distribution of proteasome during different growth states of yeasts and mammalian cells [32]. Further studies are necessary to determine if the NLS in *T*. *foetus* α-type subunits acts as a nuclear targeting sequence.

The active sites of the proteasome reside in the β-type subunits and five highly-conserved amino acids (Thr-1, Lys-33, Gly-128, Ser-129 and Gly-130) are crucial for proteolytic activity [22]. These residues are necessary not only for external peptide cleavage, but also for the autocatalytic processing of β-type subunits [33]. Thr-1 is the N-terminal nucleophile; Lys-33, Gly-128, Ser-129 and Gly-130 play a central role in catalysis, participating either indirectly by stabilising and orienting active site residues or directly as proton acceptors for the Thr-1 hydroxyl group [33–34]. All these amino acids are found in all β-type subunits from archaea; however, in eukaryotes, the proteolytic sites are present in only three subunits: β1, β2 and β5 [14, 22, 25, 33–34]. Likewise, among the seven β-type subunits from *T*. *foetus* 20S proteasome, only three (β1, β2 and β5) showed conserved residues in positions homologous to the proteolytic sites of β subunits from archaea *T*. *acidophilum* and β5 subunit from yeast *Saccharomyces cerevisiae* (Fig 3).

β1, β2 and β5 subunits are referred to as having caspase-like (C-L), trypsin-like (T-L) and chymotrypsin-like (CT-L) activities on the basis of their preference for cleaving peptides after acidic, basic or hydrophobic amino acid residues, respectively [12, 24–25, 33–35]. In yeasts, these activities are determined by specific residues located in the S1-pocket of each β subunit: Arg-45 favours the C-L activity of subunit β1, Glu-53 is responsible for the T-L activity of subunit β2, and Met-45 is attributed to the CT-L activity of subunit β5 [35]. Similarly, we found identical Arg-45 and Met-45 residues in the *T*. *foetus* subunits β1 and β5, respectively, and an aspartate residue, which is similar to glutamate, at position 53 of the β2 subunit (Fig 3), suggesting that these three subunits could have similar peptidase specificities as described for other eukaryotes.

Therefore, based on overall amino acid sequence conservation, we hypothesised that the families of *T*. *foetus* α- and β-type subunits could assume three-dimensional structures and regulatory and catalytic mechanisms similar to their respective orthologues in Archaea and eukaryotes. However, further studies are necessary to confirm this.

### Immunolocalisation of 20S proteasomes in *T*. *foetus*

In eukaryotes, proteasomes can be found in several cellular compartments, mainly the cytosol and nucleus, and are associated with the cytoplasmic face of the ER and Golgi complex. The intracellular distribution of the proteasome is a highly dynamic process that may vary according to cell type, growth conditions, cell density, cell cycle phase, cell differentiation and life cycle stage [36–37].

**Fig 4:**
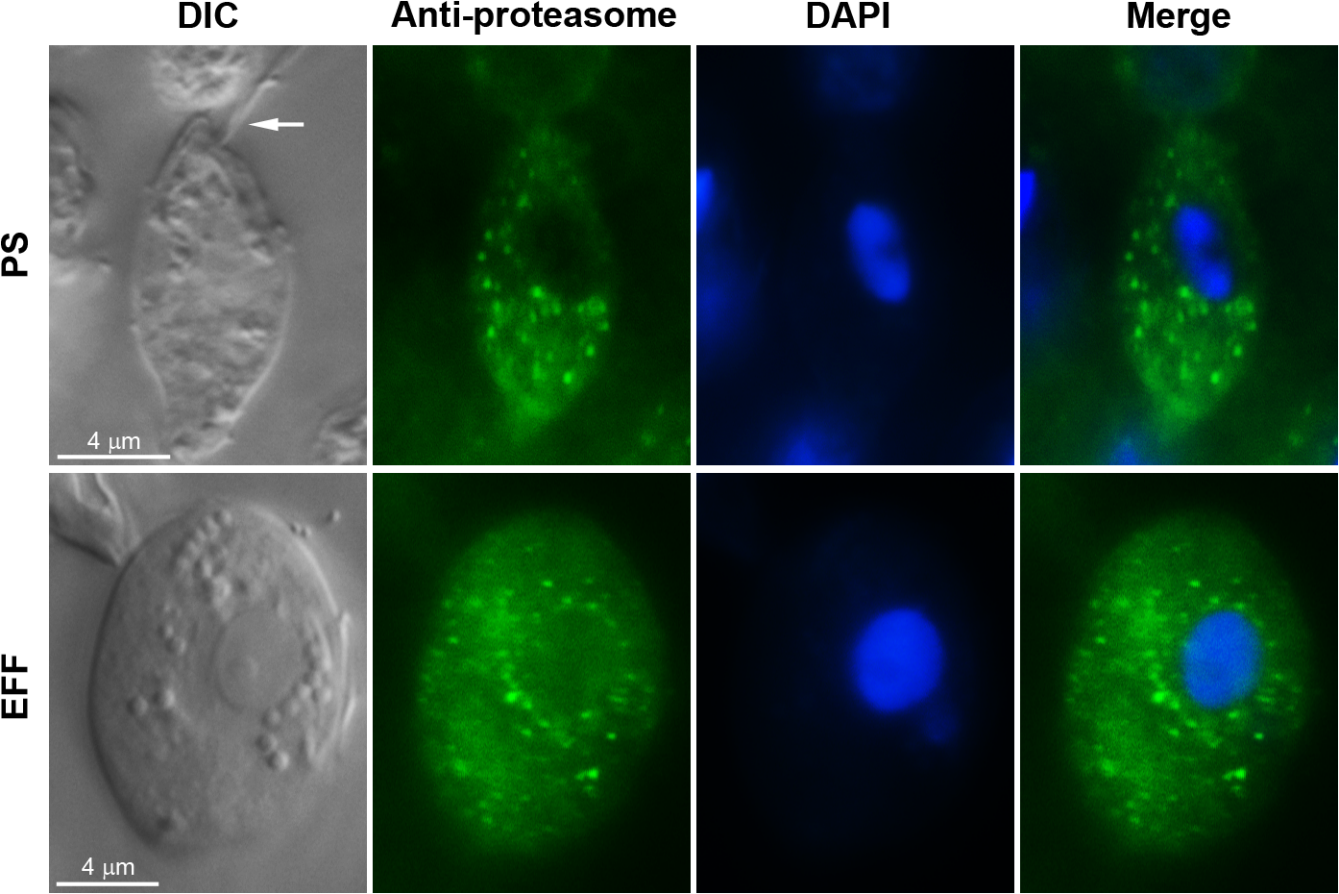
Subcellular localisation of proteasome in *T*. *foetus* using immunofluorescence. PS (first row) and EFF (second row) parasites were incubated with the polyclonal anti-*T*. *cruzi* proteasome antibody followed by DAPI staining. Column 1, DIC microscopy; column 2, the labelling pattern obtained with anti-proteasome antibody; column 3, DAPI staining; column 4, merge. In both parasite forms, the labelling is found as punctate cytoplasmic structures and in the perinuclear region. Arrow indicates the anterior flagella of the PS parasite. Bars, 4 μm.

Here, to determine the subcellular localisation of the *T*. *foetus* 20S proteasome and compare its distribution in PS and EFF parasites, we performed immunofluorescence assays using a polyclonal antibody against the *Trypanosoma cruzi* 20S proteasome that cross-reacts with the *T*. *vaginalis* proteasome [38]. Both PS and EFF *T*. *foetus* exhibited similar labelling homogeneously distributed throughout the cytosol as punctate cytoplasmic structures and in the perinuclear region, which resembled the endoplasmic reticulum localisation (Fig 4). No changes were observed in the labelling pattern of PS and EFF parasites during binary division and budding stages, respectively (S4 Fig). Although the NLS sequence was found in some subunits of the *T*. *foetus* proteasome (Fig 2), we failed to detect positive labelling in the nucleus of both parasite forms (Fig 4 and S4 Fig). Similarly, previous studies report that, in contrast to the majority of eukaryotic cells, the proteasomes of other parasitic protists, such as *T*. *gondii* [39] and *Entamoeba hystolitica* [40], are restricted to the cytosol and absent in the nucleus. However, our data did not exclude the possibility that the *T*. *foetus* proteasome could be translocated to the nucleus under an unknown specific condition. Alternatively, difficulties in epitope accessibility by the antibody could also be a reason for the negative labelling in the *T*. *foetus* nucleus. Some authors report epitope masking from interactions of proteasomes with specific proteins or post-translational modifications of some subunits [36–37]. Additional experiments are necessary to clarify this.

### Isolation and biochemical characterisation of 20S *T*. *foetus-*proteasome

**Fig 5:**
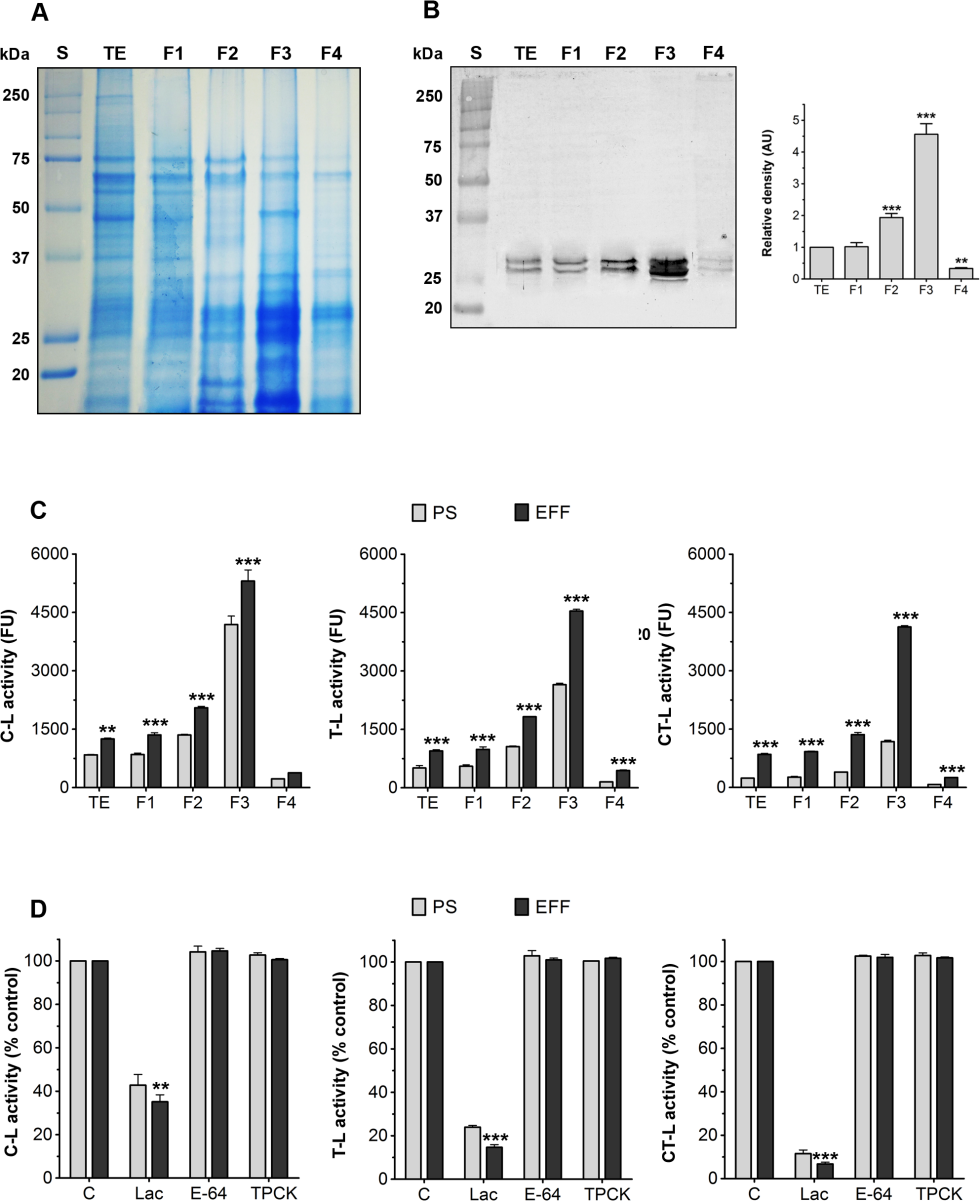
Biochemical characterisation of the fractions obtained during the *T*. *foetus* 20S proteasome isolation procedure. (A) Coomassie brilliant blue-stained 12.5% SDS–PAGE of TE, F1, F2, F3 and F4-fractions (1 X 10^9^ cells). See S5 Fig for a description of each fraction. S, molecular weight standard. Similar results were obtained for PS and EFF. (B) Immunoblot and densitometric analyses using an anti-proteasome antibody. The antibody reacted specifically with two bands near 27-kDa in each fraction. The results of densitometry were normalised to the intensity of bands in the TE fraction and are expressed as the means of relative densitometric units ± SD across three independent experiments. The proteasomal protein level increased approximately 4.5-fold in the F3-fraction. **p<0.01; ***p<0.001 compared to TE-fraction. Similar results were obtained for PS and EFF. (C) Fluorogenic substrate assay of proteasome activity of each fraction obtained from PS (light grey bars) and EFF (dark grey bars). The caspase-like (C-L), trypsin-like (T-L) and chymotrypsin-like (CT-L) activities were measured by spectrofluorometry using the fluorogenic substrates Z-LLE-AMC, Z-ARR-AMC and Z-LLL-AMC, respectively. Data are expressed as means of fluorescence units ± SD across three independent experiments performed in triplicate. The peptidase activities of proteasome from EFF were significantly higher than those from PS parasites. **p<0.01; ***p<0.001 compared to PS. In both parasite forms, the higher proteasome activity levels were found in the F3-fraction. (D) Effects of 20 μM lactacystin (Lac), 100 μM E-64 and 100 μM TPCK on the T-L, C-L and CT-L activities of F3-fractions obtained from PS (light grey bars) and EFF (dark grey bars). Data are expressed as means of relative percentage of control (without inhibitors) ± SD across three independent experiments performed in triplicate. In both forms, the peptidase activities were inhibited by lactacystin only. The proteasomal activities of the EFF were significantly more susceptible to lactacystin when compared to those of the PS. Similar results were obtained for other fractions. **p<0.01; ***p<0.001 compared to PS.

We isolated a *T*. *foetus*-proteasome-enriched fraction to characterise and compare the profile of its peptidase activities in PS and EFF parasites (Fig 5). The cytosolic proteasome-enriched fraction was obtained using an ultracentrifugation-based method (S5 Fig), as previously reported for isolation of the 20S proteasome in other eukaryotes [41–42].

**Fig 6:**
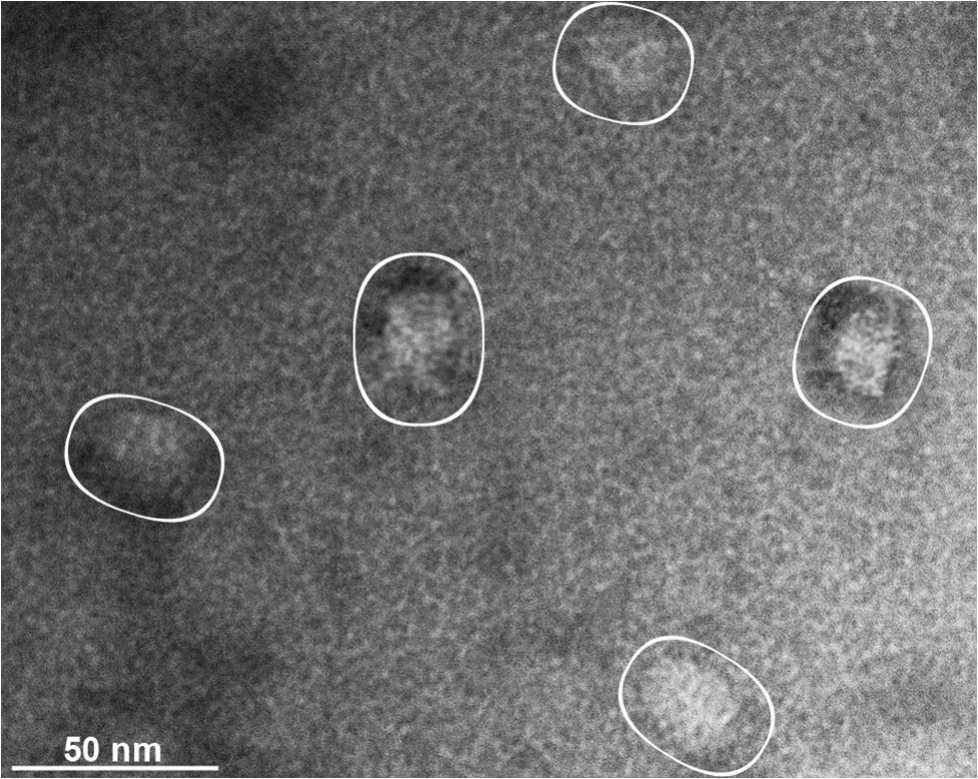
Electron micrograph of *T*. *foetus* 20S proteasome. Two μL of the F3 fraction were applied to a glow-discharge carbon-coated grid for 1 min and negatively stained with 1% uranyl acetate for 1 min. The grids were then dried and observed using transmission electron microscopy. Circles indicate examples of the typical morphology of 20S proteasome core units in a side view. Bar, 50 nm.

Analyses using SDS-PAGE showed that the F3 fraction contained an enrichment of proteins in the molecular mass range of 22 to 32 kDa (Fig 5A), consistent with the sizes of α- and β-type proteasome subunits from eukaryotes including parasitic protists [23, 43–47]. Immunoblotting revealed that the anti-*T*. *cruzi* proteasome antibody reacted with two bands near 27 kDa in each *T*. *foetus* fraction (Fig 5B). Densitometric analyses showed that, in the F3 fraction, the intensity of the bands was approximately 4.5-fold that of total cell extract (TE) fraction (Fig 5B). In addition, transmission electron microscopy (TEM) of the negatively stained samples confirmed the presence of particles in the F3 fraction (Fig 6) that resembled typical eukaryotic 20S proteasome core units [23,43,47]. Similar results were obtained for EFF *T*. *foetus* (not shown).

Fluorogenic assays using specific peptidyl substrates detected the presence of the three typical peptidase activities of eukaryotic proteasomes in all *T*. *foetus* fractions obtained from PS and EFF (Fig 5C). In both parasite forms, the higher proteasome activity levels were found in the F3-fraction (Fig 5C), validating that it was the cytosolic proteasome-enriched fraction. To rule out the possibility that the proteolytic activities could be from the action of serine, cysteine and calcium-dependent proteases commonly found in the cytosol and lysosomes, the parasite fractions were previously prepared in buffer containing EDTA and several protease inhibitors before performing fluorogenic assays. The peptidase activities of each fraction were significantly inhibited by lactacystin (20 μM), a specific proteasome inhibitor, whereas E-64 (100 μM), a cysteine protease inhibitor, and TPCK (100 μM), a serine protease inhibitor, had no effect (Fig 5D). Because it is already known that neither EDTA nor serine and cysteine proteases affect proteasome activity [48–49], our data clearly indicated that the proteolytic activities measured in the *T*. *foetus* fractions from PS and EFF were mainly a result of the proteasome.

In each fraction from PS and EFF, C-L activity was the highest, followed by T-L activity and finally, CT-L activity (Fig 5C). This result demonstrates that the *T*. *foetus* proteasome has a substrate preference profile more similar to proteasomes from other protists than from higher eukaryotes. In general, mammalian proteasomes have greater CT-L activity than T-L or C-L activities [50], whereas the reverse is found in *T*. *cruzi* [43], *Leishmania chagasi* [46], *E*. *invadens* [47], *Trypanosoma brucei* [51] and now *T*. *foetus*.

As expected, the CT-L proteasomal activity of both parasite forms was more susceptible to lactacystin (20 μM) when compared to C-L and T-L activities (Fig 5D). In eukaryotic proteasomes, lactacystin is a well-known irreversible inhibitor of CT-L activity, which binds covalently to the Thr-1 residue of subunit β5, and Met-45 contributes to this binding [35]. Moreover, lactacystin is also able to block T-L and C-L activities, albeit to a lower extent, by hydrogen-bound interactions with Thr-21, Gly-47 and Ala-49 residues of subunits β1 and β2 [35]. These amino acids were found conserved in subunits β5, β1 and β2 of *T*. *foetus* (Fig 3), suggesting that lactacystin could inhibit the parasite-proteasomal activities by a mechanism similar to that described for other eukaryotes. Further studies are necessary to demonstrate this.

Although densitometric analyses revealed that, in each fraction, the intensity of the immunoblotted bands for proteasomes between PS and EFF was similar (not shown), the three peptidase activities from EFF were significantly higher than that from PS parasites (Fig 5C). In addition, the proteasomal activities of the EFF were significantly more susceptible to lactacystin when compared to the PS (Fig 5D). Some studies report differences in the peptidase activities of proteasomes obtained from several developmental stages of *Trypanosoma* spp. [44, 49]. In *T*. *brucei*, these distinct proteolytic profiles were attributed to different proteasome subtypes between the life cycle stages of the parasite [44]. In this context, based in our data, it is tempting to speculate that the proteasomal peptidase profiles found in PS and EFF *T*. *foetus* could be from the presence of various proteasome subtypes between both parasite forms.

### Proteasome activity during the EFF induction assay

**Fig 7:**
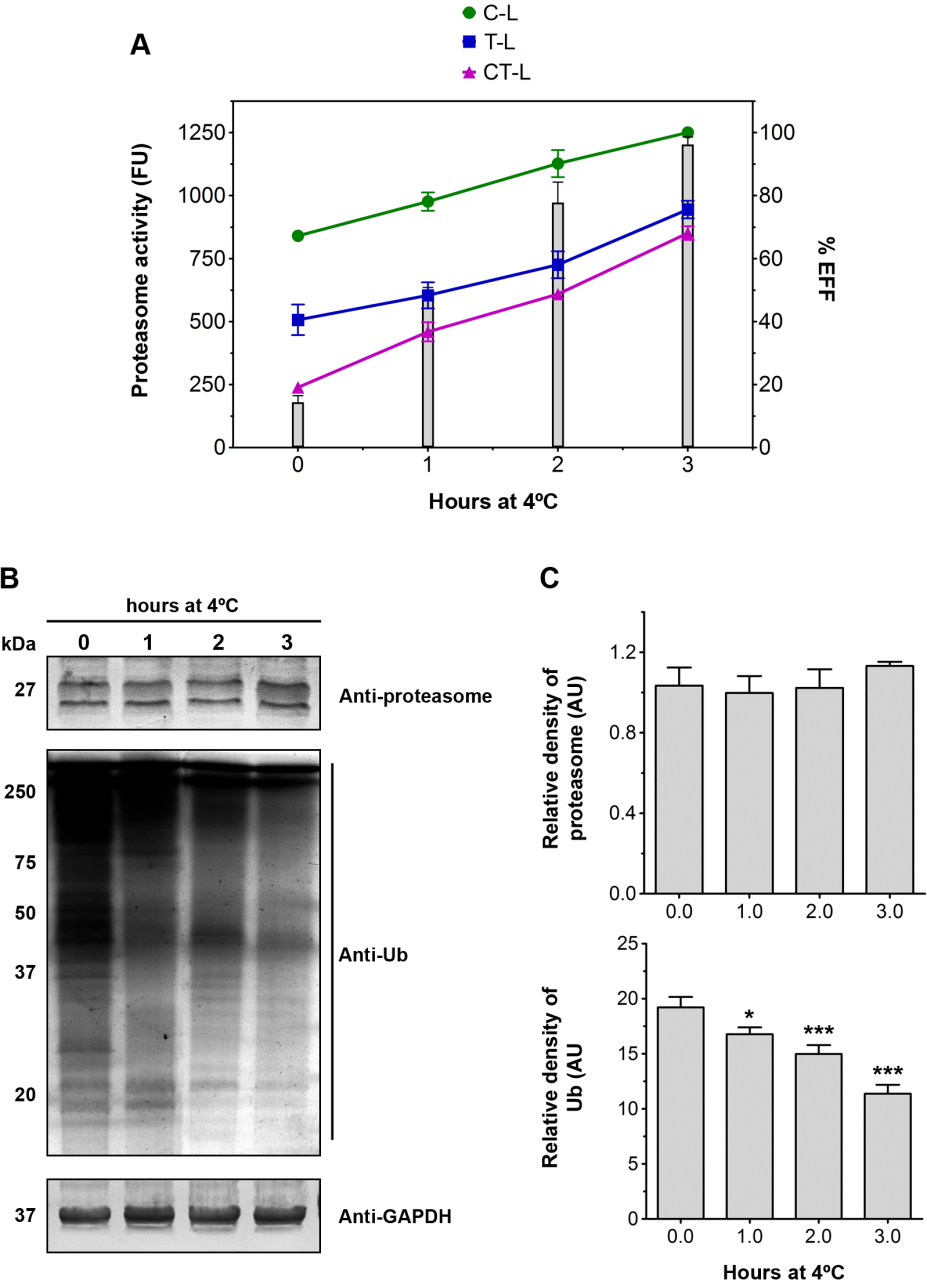
Proteasome proteolytic activity and the expression of ubiquitinated proteins during the EFF induction assay. (A) Time-course of the trypsin-like (T-L), caspase-like (C-L) and chymotrypsin-like (CT-L) proteasome during EFF induction assays. T-L (•), C-L (■) and CT-L (▲) activities were measured by spectrofluorometry using the fluorogenic substrates Z-ARR-AMC, Z-LLE-AMC and Z-LLL-AMC, respectively. The peptidase activity assays were performed using samples of the TE fractions of parasites (1 X 10^9^ cells) from different times during the EFF induction assay. Data are expressed as means of fluorescence units ± SD across three independent experiments performed in triplicate. The three peptidase activities increased over the course of EFF induction (columns). (B) Immunoblot analyses of anti-proteasome and anti-ubiquitin antibodies. GAPDH was used as a loading control. (C) Densitometric analysis of blot of anti-proteasome and anti-ubiquitin antibodies. The results are normalised to the intensity of GAPDH bands and are expressed as the means of relative densitometry units ± SD across three independent experiments. The levels of proteasomal protein levels remained unaltered, whereas the ubiquitinated proteins decreased significantly during the course of EFF induction. *p < 0.05; ***p < 0.001 compared to time 0.0.

The distinct proteasomal proteolytic activity between PS and EFF, as detected in each parasite fraction, led us to assess whether a modulation of three peptidase activities occurs during the EFF transformation. Hydrolysis of fluorogenic peptides was measured using samples of TEs of parasites obtained from standard culture conditions (t = 0.0 h) and at different times of EFF induction assay (t = 1.0, 2.0 and 3.0 h; Fig 7A). A positive correlation between C-L, T-L and CT-L activities and EFF formation was observed (Fig 7A).

To rule out the possibility that the high proteasome activity found in EFF resulted from a greater expression of proteasomes, samples were immunoblotted with anti-proteasome antibody (Fig 7B). No significant differences were detected in the proteasome levels during the EFF induction assay (Fig 7C), strongly supporting the hypothesis that the presence of different proteasome subtypes may be responsible for distinct proteolytic activities found between both *T*. *foetus* forms, as reported for bloodstream and procyclic forms of *T*. *brucei* [44]. Multiple proteasome subpopulations that differ in their proteolytic properties and substrate specificity are commonly present in eukaryotes, and their relative proportions vary according to cell type, metabolic conditions, cell cycle and differentiation phase [12, 36–37, 44]. The existence of distinct proteasome subpopulations can be attributed to following mechanisms: (a) different subunit composition of the 20S proteasome; (b) post-translational modifications of some subunits; and (c) binding of several regulatory particles to one or both ends of the 20S proteasome, e.g. 19S regulatory complex in an ATP-manner to form 26S proteasome, PA28-αβ complex in ATP independent-manner to form immunoproteasomes or PA200, an nuclear proteasome activator [12, 36–37, 44]. Additional studies are in course to investigate if these mechanisms also occur in *T*. *foetus* proteasome.

In preliminary analyses, similar to *T*. *vaginalis*, we identified in the *T*. *foetus* genome 17 homologues of a total of 19 subunits that comprise 19S regulatory complex from yeast and mammals (Rpn1, Rpn2, Rpn3, Rpn5, Rpn6, Rpn7, Rpn8, Rpn9, Rpn10, Rpn11, Rpn12, Rpt1, Rpt2, Rpt3, Rpt4, Rpt5 and Rpt6) (not shown). Only Rpn15 and Rpn13 subunits were not found. PA200 proteasome activator was also detected in the parasite genome, but PA28α and PA28β subunits were not (not shown). We tried to detect 19S regulatory complex in *T*. *foetus* by immunofluorescence and immunoblotting using monoclonal or polyclonal antibodies against mammal, yeast or *T*. *cruzi* 19S subunits, but no labelling was detected. In addition, we failed to find the presence of particles that resemble 19S cap using TEM of the negatively stained samples. Because the regulatory complexes are very labile, become dissociated during the purification procedure and do not withstand standard chromatographic methods or exposure to high ionic strength buffers [42, 44, 52], experimental procedures are still being assessed by our group to investigate the presence of 26S proteasome and other regulatory particles in *T*. *foetus*.

Here, all fluorogenic assays were performed in the presence of ATP. In the absence of ATP, the peptidase activities were approximately five-fold lower (not shown). Because ATP-dependent proteasomal proteolysis in eukaryotic cells is coupled with the ubiquitination process through removal of ubiquitinated substrates [12–14,24–28,33–37], we investigated whether there was a correlation between the increase in the proteasome activities and the levels of ubiquitinated proteins during the EFF induction assay. To evaluate this, TEs of parasites obtained from different times of EFF induction assay (t = 0.0, 1.0, 2.0 and 3.0 h) were immunoblotted with a monoclonal anti-Ub antibody (Fig 7B). Densitometric analyses showed that the levels of Ub-protein conjugates decreased in the time-course of the EFF transformation assay (Fig 7C). This is consistent with a previous study that demonstrated a reduction in the expression levels of Ub and poly-Ub in iron-depleted induced EFF of *T*. *vaginalis* [53]. Our data indicated that at least a part of the high proteasome activity found in EFF parasites could be responsible for the reduced level of ubiquitinated proteins at the end of the induction assay, suggesting the presence of a Ub/26S proteasome complex in *T*. *foetus* similar to that found in other eukaryotes, including parasitc protists such as *Leishmania mexicana* [45], *T*. *cruzi* [48] and *Plasmodium falciparum* [54]. Studies are on course to identify the Ub-protein conjugates that vary their ubiquitination status during EFF induction.

Although this study suggests a correlation between EFF transformation, increases in proteasome activity and reduced levels of Ub-protein conjugates, these results should be interpreted with caution for several reasons. First, the proteolytic activity assays were measured *in vitro* using a cell-free system, different from *in vivo* settings where the heterogeneity and plasticity of proteasomes are dynamically controlled to meet specific subcellular needs or to respond to stress or other stimuli. In this context, studies have demonstrated that, in cell homogenate or during cell fractionation, some active proteasome complexes from distinct subcellular compartments can lose functional integrity, changing their proteolytic activities or even becoming inactivated [36, 42]. Second, the protein ubiquitylation system also acts as signalling pathways, e.g. for protein importation, which does not involve the proteasomal degradation pathway [49, 55]. Finally, we did not investigate the levels of oxidised proteins and the ATP-independent proteasomal proteolysis during the EFF induction assay. It is known that the proteasomal system is the major proteolytic pathway responsible for the removal of oxidised proteins by an ATP/Ub-independent mechanism [49, 56]. Studies report that cold exposure may provoke intense oxidative damage in eukaryotic organisms, resulting in the increase of proteasome proteolytic activity to protect the cells against the elevated level of oxidised proteins [57–59]. Recently, Fang et al. demonstrated that cold stress induces an increase in the proteasome activity of *T*. *vaginalis* [60]. Because EFF transformation was induced by low temperature, our data do not exclude the possibility that a part of the high proteasome activity found in EFF parasites could be from cold stress and involve oxidised protein degradation. Studies are on course to determine the levels of oxidised proteins in cold-induced EFF *T*. *foetus* and whether there is a correlation with ATP-independent proteasomal proteolysis during parasite transformation.

### Effects of lactacystin on growth, cell cycle and ultrastructure of PS *T*. *foetus*

Lactacystin is a useful compound for determining the physiological roles of proteasome in eukaryotic cells, including parasitic protists [43, 45–49, 61–64]. Here, lactacystin was used to investigate whether proteasomal proteolysis is involved in biological events of PS *T*. *foetus*, such as cell growth and replication. Because this compound may affect other proteases in addition to proteasome activity, such as calpain and cathepsins [35], E-64d (50 μM) was used as a control to rule out the possibility that our results were caused by an unspecific inhibitory action of lactacystin.

**Fig 8:**
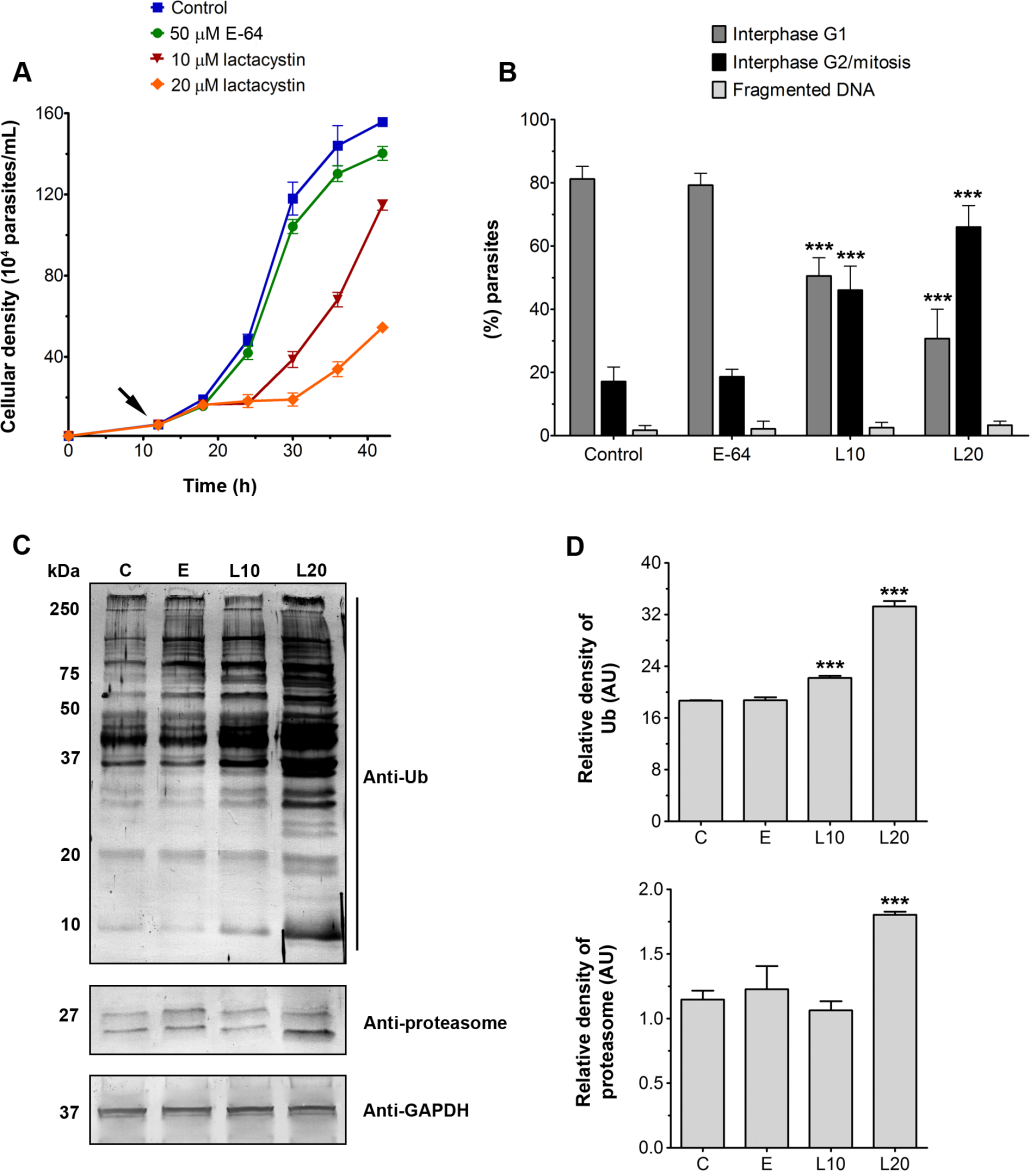
Effects of lactacystin on growth, cell cycle and ubiquitinated protein levels of PS *T*. *foetus*. (A) Growth curve of parasites. The parasites were initially cultured for 12 h at 37°C (initial inoculum: 1x10^4^ parasites/mL). After this period (arrow), 50 μM E-64d,10 and 20 μM of lactacystin were added to the culture medium and parasites were incubated for up to 30 h at 37°C. The cell growth was calculated after 6, 12, 18, 24 and 30 h of incubation. Untreated parasites were used as a control. Values are expressed as the means ± SD across three independent experiments, each performed in triplicate. (B) Analyses of the DNA content of *T*. *foetus* after treatment with 50 μM E-64d, and 10 (L10) and 20 μM (L20) of lactacystin for 12 h. Data acquisition and analysis were performed using a flow cytometer. Values are expressed as means ± SD across three independent experiments. Note that lactacystin arrests the *T*. *foetus* cell cycle in the G2/mitosis phases. ***p < 0.001 compared to control. (C) Immunoblot analyses of anti-ubiquitin and anti-proteasome antibodies. C, control; E, parasites treated with 50 μM E-64d for 12 h; L10 and L20, parasites treated with 10 μM and 20 μM lactacystin for 12 h, respectively. GAPDH was used as loading control. (D) Densitometric analysis of blot of anti-ubiquitin and anti-proteasome antibodies. The results are normalised to the intensity of GAPDH bands and are expressed as the means of relative densitometry units ± SD across three independent experiments. The levels of ubiquitinated and proteasomal proteins increased significantly when the parasites were treated with 20 μM lactacystin (L20) for 12 h. ***p < 0.001 compared to control.

To evaluate the effects of lactacystin on *T*. *foetus* growth, parasites were initially cultured for 12 h at 37°C. After this period, different concentrations of compound were added to the culture medium and the number of cells/mL was calculated after 6, 12, 18, 24 and 30 h of incubation (Fig 8A). Parasite growth was inhibited by lactacystin in a dose-dependent manner. No inhibitory effects were observed in cultures treated with up to 5 μM lactacystin (not shown). However, treatment with 10 and 20 μM lactacystin completely inhibited the culture growth for 12 h and 18 h, respectively (Fig 8A). After these times, *T*. *foetus* growth was restored, but at a lower rate when compared to untreated parasites (Fig 8A). Based on these results, subsequent experiments with 10 and 20 μM of lactacystin for 12 h were performed. Analyses of DNA content per cell using a flow cytometer revealed that lactacystin arrested the *T*. *foetus* cell cycle in the G2/mitosis phases in a dose-dependent manner (Fig 8B). In addition, lactacystin treatment resulted in an accumulation of Ub-protein conjugates in *T*. *foetus*, as shown by immunoblot analyses (Fig 8C and 8D), indicating that the compound penetrated the parasite and inhibited proteasome peptidase activity, as observed in other eukaryotic cells [48, 53, 65–66]. E-64d did not significantly affect culture growth, cell replication or the level of ubiquitinated proteins in the parasites (Fig 8A–8D), and *T*. *foetus* viability remained unaltered during all treatments. Taken together, these data strongly suggest that the Ub-proteasome system is required for *T*. *foetus* cell cycle and growth, as reported for other protists, such as *Leishmania* spp. [45–46], *T*. *cruzi* [61], *Entamoeba* spp. [62], *Plasmodium* spp. [54, 63] and *T*. *gondii* [64].

Interestingly, our immunoblot analyses also showed that the expression of proteasome bands increased significantly when the parasites were treated with 20 μM lactacystin for 12 h (Fig 8C and 8D). In mammalian cells, the synthesis of proteasome is up-regulated at the transcriptional level in response to proteasomal activity inhibition [66–67]. It is tempting to speculate that a similar proteasome recovery pathway could occur in *T*. *foetus*. The increase of proteasomes could help to explain, at least in part, why *T*. *foetus* growth was restored after treatment with 20 μM lactacystin for 18 h (Fig 8A). However, additional studies are necessary to investigate this hypothesis.

**Fig 9:**
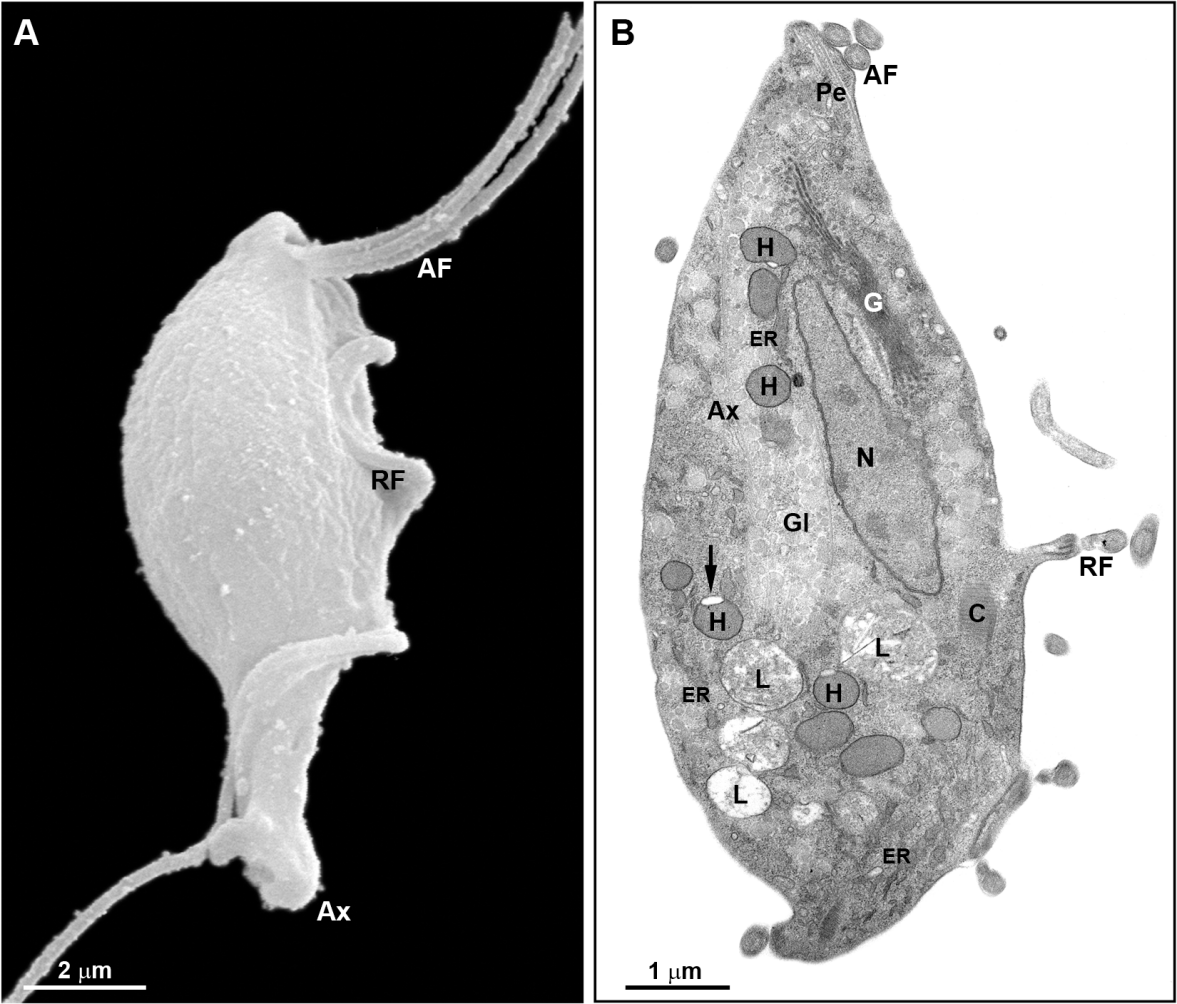
Fine structure of untreated PS *T*. *foetus*. (A) SEM. Parasite exhibits three anterior flagella (AF) and one recurrent flagellum (RF) with a distal free end. The axostyle (Ax) tip is visible. (B) TEM of a longitudinal section of *T*. *foetus*. The parasite displays one anterior nucleus (N), pelta (Pe), axostyle (Ax), a well-developed Golgi complex (G), hydrogenosomes (H) with only one peripheral vesicle (arrow), lysosomes-like structures (L) and endoplasmic reticulum (ER). Bars: A, 2 μm; B, 1μm.

**Fig 10:**
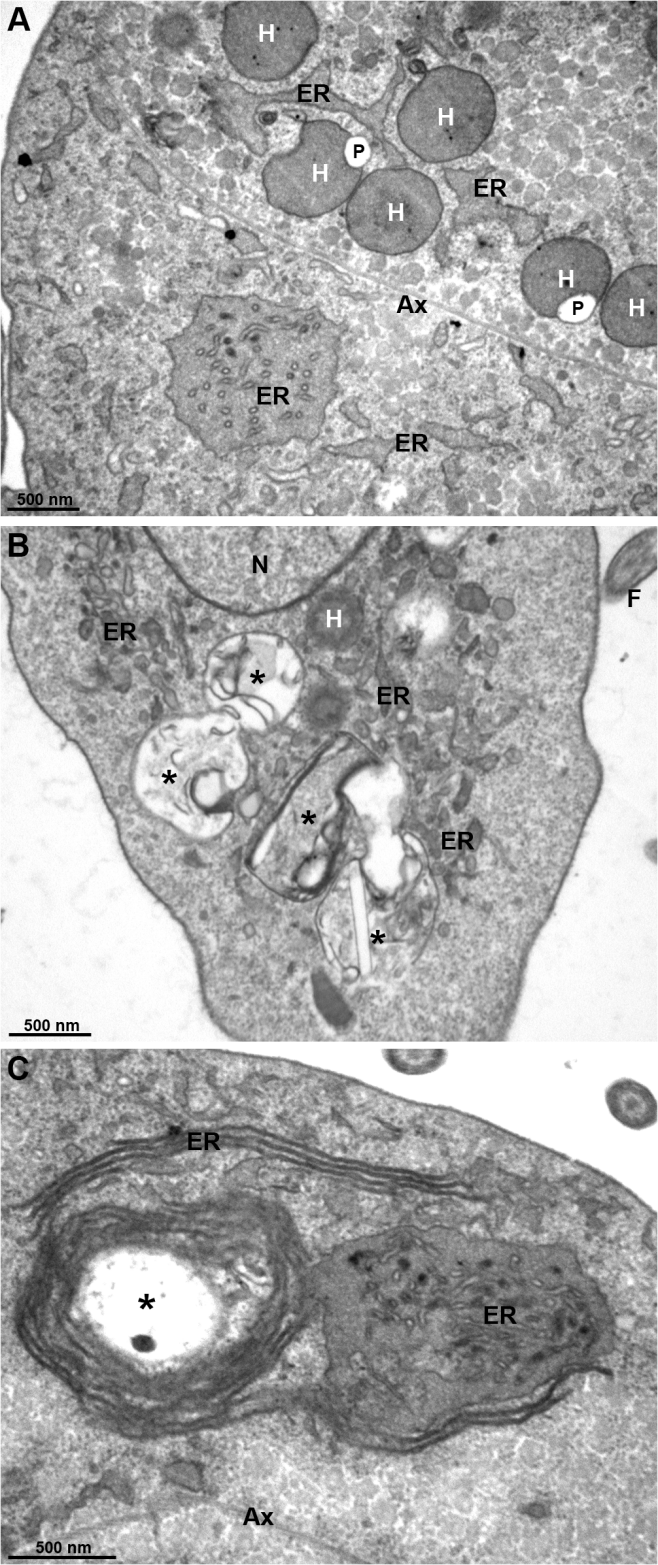
TEM of lactacystin-treated PS *T*. *foetus*. Parasites were incubated with 20 μM lactacystin for 12 h. The parasites exhibit uncommon enlarged endoplasmic reticulum (ER)-derived membranes and concentric membrane whorls (asterisks), which resemble autophagy vacuoles. Other structures, such as hydrogenosomes (H), nucleus (N) and axostyle (Ax) remain unaltered. Bars, 500 nm.

To determine the effects of lactacystin on the morphology and fine structure of *T*. *foetus*, PS parasites treated with 20 μM lactacystin for 12 h were observed using scanning (SEM) and transmission (TEM) electron microscopy. Untreated PS *T*. *foetus* displayed the typical morphology without ultrastructural alteration (Fig 9), and lactacystin-treated parasites did not exhibit apparent alteration of their external morphology (S6 Fig). However, the proteasome inhibitor provoked the appearance of an uncommon enlarged ER and concentric membrane whorls, which resembled autophagic vacuoles (Fig 10). Similar ultrastructure alterations were also found in *T*. *gondii* after lactacystin treatment [64]. E-64d, an inhibitor of some calpain and cathepsin proteases, did not induce morphological changes found in lactacystin-treated *T*. *foetus*, however, alterations in lysosome-like structures and hydrogenosomes were observed (S7 Fig). Therefore, our data suggest that the effects of lactacystin on the ultrastructure of *T*. *foetus* could be provoked by a specific inhibition of proteasome activity.

In higher eukaryotes, proteasomes associated with ER are involved in a constitutive process for protein quality control, known as the ER-associated degradation (ERAD) pathway, which consists of the selective retrograde transport of structurally abnormal or misfolded proteins from the ER back to the cytosol, associated subsequently with their ubiquitination and proteasome-dependent degradation [37]. Some studies have demonstrated the presence of the ERAD system in apicomplexan protists, and this pathway could be blocked by action of proteasome inhibitors [54–55]. Although the functional dynamics of ER in *T*. *foetus* remain unknown, it is tempting to speculate, based on our data and assuming conserved functional characteristics between ER, that the inhibition of proteasomal activity by lactacystin could impair an ERAD-like system in *T*. *foetus*, blocking the normal disposal of abnormal or misfolded proteins, and this would in turn lead to development of large amounts of ER membranes and autophagy-like bodies in the parasite. However, studies are necessary to determine the ER functions and the existence of an ERAD-like pathway in *T*. *foetus*.

Lactacystin-treatment did not affect *T*. *foetus* viability and the effects of compound on growth, cell cycle and ultrastructure of PS parasites described here were reversible after 12 h and 18 h of incubation with 10 and 20 μM of lactacystin (not shown). Because a significantly high concentration of lactacystin was required to completely inhibit the parasite growth, probably due to lower permeability or poor efficacy of the compound in cell systems [35, 45–46], we tried to complement our data with more potent, permeable and specific proteasome inhibitors, such as epoxomicin and bortezomib (S8 Fig).

Although epoxomicin and bortezomib possess high specificity for the 20S core particles and do not inhibit other proteases like calpain, trypsin, chymotrypsin, papain or cathepsins [35], we found that both compounds were not a good choice to investigate the physiological roles of proteasome in *T*. *foetus*. Treatment with epoxomicin and bortezomib irreversibly inhibited the culture growth and provoked a trichomonacidal effect in a dose-dependent manner (S8A and S8B Fig). Ultrastructural analyses of PS parasites treated with 0.01 μM epoxomicin and 0.1 μM bortezomib for 6 h revealed the presence of several alterations indicative of cell death, including the appearance of wrinkled or rounded cells with externalised flagella, membrane blebbing, cell lysis, intense cytosolic and nuclear vacuolization, cytoplasmic disintegration and abnormal Golgi reduction (S8C–S8F Fig). These effects were irreversible even after drug withdrawal (not shown). No effects on cell growth, viability and morphology were observed in cultures treated with concentrations lower than 0.01 μM epoxomicin and 0.1 μM bortezomib (not shown). It is well known in the literature that proteasome inhibitors can induce antiproliferative effects and cell death in protist and mammal cells [35, 68–71]. However, among compounds assessed here, only lactacystin did not induce irreversible effects, loss of viability and cell death in *T*. *foetus*.

### Effects of lactacystin on EFF *T*. *foetus* transformation

**Fig 11:**
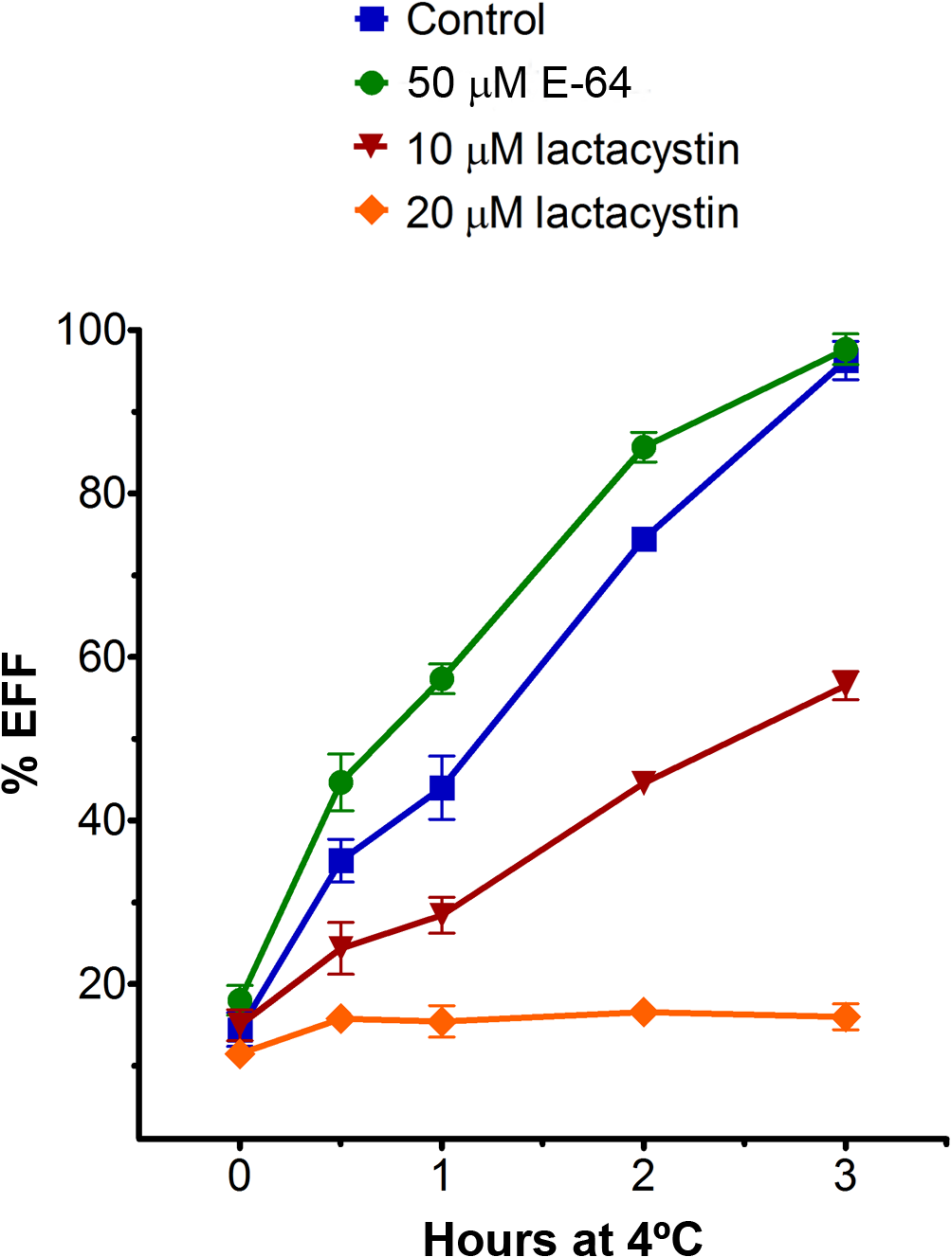
Effects of lactacystin on *T*. *foetus*-EFF transformation. The parasites were treated with 50 μM E-64 and 10 μM or 20 μM lactacystin for 12 h, washed, resuspended in culture medium in the absence of lactacystin or E-64 and submitted to the EFF induction assay. Untreated parasites were used as a control. The EFF percentage was determined from counts of at least 1,000 parasites per sample at several time points (0, 0.5, 1, 2 and 3 h) using light microscopy. The EFF transformation was completely inhibited when the parasites were treated with 20 μM lactacystin.

The biological mechanisms that regulate the transformation of the polarised PS form to the EFF have not been elucidated yet. The EFF *T*. *foetus* may be naturally found in preputial smegma from bulls, but *in vitro* EFF formation is associated with stress conditions. Surprisingly, lactacystin treatment did not induce EFF transformation in PS parasite cultures (S6A Fig). Because proteasomes are required for the morphological transitions in some protozoa such as, *Trypanosoma* spp. [34, 43–44, 48–49, 61], *Leishmania chagasi* [46], *Entamoeba* spp. [47, 62] and *Plasmodium* spp. [63], we evaluated whether lactacystin affects EFF *T*. *foetus* formation during cold-induction assay. Parasites were treated with 10 μM or 20 μM lactacystin for 12 h, washed, resuspended in culture medium in the absence of lactacystin and submitted to the EFF induction assay (Fig 11). The EFF transformation occurred partially when the parasites were treated with 10 μM lactacystin, and the treatment with 20 μM lactacystin completely inhibited the EFF formation when compared to untreated *T*. *foetus* during induction assay (Fig 11). E-64d slightly increased the EFF transformation rate (Fig 11). The inhibitory effect of lactacystin on EFF formation was reversible after 12 h and 18 h of incubation with 10 and 20 μM of compound, respectively (not shown). Taken together, our data strongly suggest that the proteasome activity may play a physiological role during the transformation of PS *T*. *foetus* into EFF, similar to that reported for life stage transformation in other protists.

## Conclusions

This study contributes to a better understanding of the trichomonad’s cell biology. Our data confirm that *T*. *foetus* has a 20S proteasome with predicted amino acid sequences and a profile of peptidase activities similar to other eukaryotes. To our knowledge, this work sheds some light on the Ub-proteasome pathway in cell cycles and EFF transformation in *T*. *foetus*. The identification of possible targets for degradation by proteasomes may be useful for revealing the mechanisms that underlie the parasite’s replication and EFF formation and can provide a promising target for chemotherapy.

## Materials and Methods

### Chemicals and substrates

All compounds were purchased from E. Merck (D-6100 Darmsttadt, Germany) or Sigma-Aldrich Chemical Co. (St. Louis, MO). Lactacystin (stock solution: 5 mM) and E-64d (stock solution: 30 mM) were dissolved in deionised distilled water. TPCK (stock solution: 50 mM), Epoxomicin (stock solution: 1 mM) and Bortezomib (stock solution: 1 mM) were solubilized in DMSO. The fluorogenic proteasome substrates Z-LLL-AMC, Z-ARR-AMC and Z-LLE-AMC were also prepared in DMSO at a stock concentration of 5 mM. All reagents were stored at -20°C until use.

### Parasite culture

The CC09-1 strain was obtained by Dr. C. M. Campero (Instituto Nacional de Tecnología Agropecuaria, Buenos Aires, Argentina) and Dr. A. Martínez (Laboratorio Azul, Buenos Aires, Argentina) from the genital tract of a bull naturally infected with *T*. *foetus* and axenized in 2009, as previously published [6]. The parasites were cultivated in a trypticase, yeast extract and maltose (TYM) medium [72] supplemented with 10% foetal bovine serum. *T*. *foetus* was grown in Pyrex culture tubes (O.D. × L: 16 mm × 150 mm), containing 20 mL of TYM medium (initial inoculum: 1x10^4^ parasites/mL), for 30 h at 37°C, which corresponds to the logarithmic growth phase. The viability of the parasites was checked before and after each assay using the trypan blue dye exclusion method (0.4% in sterile PBS).

### DNA extraction

Parasites (1x10^8^ cells) were harvested by centrifugation, washed three times with PBS (pH 7.2) and immediately resuspended in ethanol (96%, vol/vol). Afterwards, the total genomic DNA was purified with Wizard Genomic DNA Purification Kit (Promega, USA) according to the manufacturer´s instructions. DNA concentration, purity, and the overall integrity were checked using a spectrophotometer (O.D.260 nm/O.D.280nm ratio) and by agarose gel electrophoresis.

### Pyrosequencing and sequence analysis

Genome sequencing was performed at Computational Genomics Unity Darcy Fontoura de Almeida (UGCDFA) of the National Laboratory of Scientific Computation (LNCC) (Petrópolis, RJ, Brazil). Approximately 500 ng of total *T*. *foetus* DNA was sequenced using 454-pyrosequencing methodology [17] following the GS-FLX Titanium series protocols (Roche Applied Science). All titrations, emulsion PCR and sequencing steps were carried out according to the manufacturer's instructions. The shotgun library was constructed on one of the two regions of a 70×75 PicoTiterPlate using the Genome Sequencer FLX System (Roche). Before sequence analysis and assembly, artificially replicated sequences that are generated as an inherent artefact of 454-based pyrosequencing were identified and removed using the Replicates software [73].

Fourteen protein sequences of the *T*. *vaginalis*-20S proteasome subunits were selected from the KEGG ORTHOLOGY (KO) database (http://www.genome.jp/kegg-bin/show_module?tva_M00340). The selected sequences were compared to reads of *T*. *foetus* using tBLASTN 2.2.24 software. An E-value cut-off threshold of 1e−50 was applied to define a set of significant reads to reconstruct each protein sequence. Each protein sequence was reconstructed with the counterpart set of reads selected using the software Newbler 2.6. The Consed 20.0 software was used to align and select the contigs. The existence of the gene was then confirmed using the ORF Finder software (http://www.ncbi.nlm.nih.gov/gorf/gorf.html) and BLASTp 2.2.24 software.

### Nucleotide sequence accession numbers

The sequences of *T*. *foetus* proteasome were deposited in GenBank (http://www.ncbi.nlm.nih.gov/genbank/) with the accession numbers KF428747 to KF428760.

### Sequence alignment and conserved domains database

The amino acid sequences of the 20S proteasome subunits from *T*. *vaginalis*, *T*. *cruzi*, *D*. *discoideum*, *S*. *cerevisiae*, *A*. *thaliana*, *C*. *elegans*, *D*. *melanogaster* and *H*. *sapiens* were retrieved from the KO database (http://www.genome.jp/kegg-bin/show_module?map=M00340). The protein sequences of the *T*. *foetus*-20S proteasome are from the present work. All sequences were compared against each other using BLAST (http://blast.ncbi.nlm.nih.gov).

Multi-FASTA putative orthologous and paralogous files (S1 and S2 Figs; S1 Table) were used as input for multiple alignments using CLUSTALw algorithm with default parameters. Alignments were visualized and edited using the Genedoc package version 2.7 (http://www.psc.edu/biomed/genedoc/). The phylogenetic tree was constructed by the neighbor-joining method based on the alignment. The distance matrix was obtained by calculating p-distances for all pairs of sequences. Gaps were excluded using the pairwise-deletion option. Branch points were tested for significance by bootstrapping using 1,000 replications. The MEGA version 5.2.2 software [74] was used to perform these results as an unrooted dendrogram.

The conserved domains and active sites of the predicted amino acids sequences of *T*. *foetus*-20S proteasome subunits were determined using the NCBI CD-Search (http://www.ncbi.nlm.nih.gov/Structure/cdd/wrpsb.cgi) software with default parameters.

### EFF induction assay

To induce the EFF transformation cultures of *T*. *foetus* grown for 30 h at 37°C in TYM medium were cooled to 4°C for up to 3 h, without changing the medium during this period, as previously described [7]. Parasites grown for 30 h under standard conditions were used as control. The EFF percentage was determined from counts of at least 1,000 parasites per sample after several time points using light microscopy. Parasites grown for 30 h under standard conditions were used as controls. The percentage of PS and EFF was estimated before and after every experimental procedure that was performed throughout this study. The viability of the parasites was checked the trypan blue dye exclusion method.

### Immunofluorescence microscopy

PS and EFF were fixed with 4% paraformaldehyde in phosphate buffer (pH 7.2), washed in PBS (pH 8.0) and allowed to adhere to poly-L-lysine-coated glass coverslips. The parasites were permeabilized with 0.1% Triton X-100 for 10 min and blocked with 50 mM ammonium chloride and 3% BSA/PBS for 15 min each step. Next, the parasites were incubated overnight at 4°C with the polyclonal anti-*T*. *cruzi* proteasome antibody (kindly provided by Dr. Jorge González—University of Antofagasta, Chile), diluted 1:50 in 1% BSA/PBS. Afterwards, the samples were incubated with an anti-rabbit IgG Alexa-488-conjugated secondary antibody (Life Technologies, USA), diluted 1:100 in 1% BSA/PBS, for 40 min at room temperature. Parasites were also stained with 5 μg/ml DAPI for 5 min. The samples were observed using a Zeiss Axiophot II fluorescent microscope (Zeiss, Germany) using a 100X, N.A. 1.3 objective lens. The images were randomly acquired with a high resolution digital camera (AxioCam MRc5, Zeiss, Germany).

### Preparation of *T*. *foetus* cell extract and cytosolic proteasome-enriched fraction

Parasites (1 X 10^9^ cells) grown for 30 h under standard conditions and obtained from different times of EFF induction assay (1, 2 and 3 h) were harvested by centrifugation and washed three times in cold 10 mM Tris–HCl buffer (pH 7.4), containing 0.25 M sucrose and 2 mM MgCl_2_. Afterwards, the parasites were resuspended in lysis buffer (10 mM Tris-HCl, pH 7.4, 0.25 M sucrose, 2 mM EDTA, 2 mM DTT, 2 mM MgSO_4_, 150 mM KCl, 30% glycerol) containing a protease inhibitor cocktail (Sigma—Cat. no. P-2714). Next, the parasites were disrupted with 300 strokes of a Potter-type homogeniser on ice. The cell homogenates were then pre-cleared by centrifugation (1,000 x g for 10 min at 4°C) and the supernatants (total cytosolic extract—TE) were collected (S5 Fig).

To obtain the proteasome-enriched fraction, the TEs of parasites under standard culture conditions and at the end of EFF induction assay were submitted to three sequential ultracentrifugation steps (S5 Fig) using a Beckman Type 65 rotor (Beckman Coulter, USA). The first step was at 12,000 x g for 20 min, the resulting supernatant fraction (F1) was then centrifuged at 150,000 x g for 1 h, and the supernatant from this (F2) was centrifuged at 150,000 x g for 6 h (S5 Fig). Afterwards, the resulting pellet (F3), enriched in cytosolic proteasomes, was collected and resuspended in 100 μL of buffer (10 mM Tris-HCl, pH 7.4, 2 mM EDTA, 2 mM DTT, 2 mM MgSO_4_, 150 mM KCl, 30% glycerol and protease inhibitor cocktail). The supernatant fraction of the last step (F4) was also collected (S5 Fig). All centrifugation procedures were performed at 4°C.

The protein content of TEs and each fraction described here was determined by the Lowry method using bovine serum albumin as standard.

### SDS-PAGE analysis

The TE, F1, F2, F3 and F4 fractions of the parasites (1 X 10^9^ cells) obtained from both standard culture conditions and at the end of EFF induction assays were analysed by SDS-PAGE using 12.5% gradient gel. The same amount of protein (20 μg) from all of the samples was boiled and applied to the gel. Kaleidoscope Pre-stained Standard (Bio-Rad, Brazil) was used as molecular weight standard. The gels were stained with Coomassie Brilliant Blue G (CBB-G-250). The images were acquired with a digital camera (Fuji FinePix S3300, FujiFilm, Japan).

### Detection of proteasomes using Western blot analysis

Samples of the TEs (standard culture conditions and different times of EFF induction assay) and fractions obtained by differential ultracentrifugation were separated by SDS-PAGE, as described above, and transferred to a polyvinylidene fluoride (PVDF) membrane (Millipore Corporation, USA). The PVDF membrane was blocked with 5% fat-free milk in TBS-T buffer (150 mM NaCl, 10 mM Tris-HCl, pH 8,0, supplemented with 0.1% Tween-20) and incubated with the polyclonal anti-*T*. *cruzi* proteasome antibody, diluted 1:1000 in the same blocking solution, for overnight at 4°C. The specifically bound primary antibodies were detected using a horseradish peroxidase-conjugated goat anti-rabbit IgG antibody, diluted 1:5,000 in the blocking solution and incubated for 1 h at room temperature. Blots were washed in TBS-Tween and developed using the DAB Enhanced Liquid Substrate system ECL (Sigma-Aldrich Chemical Co., St. Louis, MO), according the manufacturer’s instructions. The images were acquired with a scanner (HP G4050, Hewlett-Packard, Brazil). In some experiments a monoclonal anti-*T*. *cruzi* GAPDH antibody (dilution 1:1000) (kindly provided by Dr. José Roberto Meyer Fernandes—Federal University of Rio de Janeiro, Brazil) was used as loading control. The protein levels were quantified by densitometry analyses using GelQuantNET software (http://biochemlabsolutions.com/GelQuantNET.html) and were expressed in relative densitometry units. When the experiments were performed using the fractions from differential ultracentrifugation, the results were normalized to the intensity of TE bands. When the experiments were carried out with TEs only, the results were normalized to the intensity of GAPDH bands for each corresponding sample in the gel lane.

### Measurement of the peptidase activity of proteasomes

The fluorogenic substrates Z-ARR-AMC, Z-LLE-AMC and Z-LLL-AMC were used to measure the T-L, C-L and CT-L proteasome activities, respectively. Assays were carried out in a total volume of 240 μL of reaction medium containing 100 μg of the TEs (standard cultures conditions and different times of EFF induction assay) or fractions obtained by differential ultracentrifugation, 50 μM fluorogenic substrates and buffer (50 mM Tris-HCl, pH 7.5, 5 mM MgCl_2_, 1 mM DTT and 1 mM ATP). The samples were then incubated at 37°C for 30 min in the presence or the absence of 20 μM lactacystin, 100 μM E-64 or 100 μM TPCK. As blank, the reaction medium was incubated in the absence of TEs and ultracentrifugation fractions. The reaction was stopped by adding 2 mL of cold ethanol. Afterwards, the reaction tubes were centrifuged at 1,500 x g for 10 min and the peptidase activities were determined by fluorimetric quantification of supernatant with a spectrofluorophotometer at excitation and emission wavelengths of 380 nm and 440 nm, respectively. The results are expressed as fluorescence units.

### Detection of ubiquitinated proteins

Samples of the TEs (standard cultures conditions and different times of EFF induction assay—1 X 10^9^ cells) were separated by 12.5% or 10% SDS-PAGE, transferred to a PVDF membrane and blocked with 5% fat-free milk in TBS-T buffer, as described above. The Ub-protein conjugates were then detected using the monoclonal anti-bovine Ub antibody (clone P4D1, Santa Cruz Biotechnology), diluted 1:1000, overnight at 4°C, followed by incubation of a horseradish peroxidase-conjugated goat anti-mouse IgG antibody, diluted 1:5,000, for 1 h at room temperature. Blots were developed using the DAB Liquid Substrate and the images were acquired as mentioned above. The monoclonal anti-*T*. *cruzi* GAPDH antibody (dilution 1:1,000) was used as loading control. The relative changes in Ub-protein conjugates levels during EFF induction assays were determined by densitometry analyses as described above. The results were normalized to the intensity of GAPDH bands for each corresponding sample in the gel lane and were expressed in relative densitometry units.

To detect the lactacystin-induced accumulation of Ub-protein conjugates, the standard cultures of parasites were incubated with 10 μM or 20 μM lactacystin for 12 h, as mentioned below. Untreated parasites or cultures treated with 50 μM E-64d for 12 h were used as controls. Afterwards, the parasites (1 X 10^6^ cells) were harvested by centrifugation, washed and the TEs were collected as described above. Next, samples of the TEs (20 μg) were separated by 12.5% SDS-PAGE, transferred to a PVDF membrane and the Ub-protein conjugates were detected as previously mentioned.

### Effects of proteasome inhibitors on the parasite growth

The parasites were cultured in TYM medium for 12 h at 37°C (initial inoculum: 1x10^4^ parasites/mL). After this period, several concentrations of lactacystin (1, 5, 10 and 20 μM), epoxomicin (0.01, 0.1 and 1 μM) and bortezomib (0.1, 0.5 and 1 μM) were added to the culture medium and the parasites were incubated for up to 30 h at 37°C. The number of parasites/mL was calculated after 6, 12, 18, 24 and 30 h of incubation using a Neubauer hemocytometer. The viability of the parasites was checked using the trypan blue dye exclusion method (0.4% in sterile PBS). Untreated parasites or cultures treated with 50 μM E-64d were used as controls.

### Effects of lactacystin on the parasite cell cycle

The parasites were incubated with 10 μM or 20 μM lactacystin for 12 h, as previously described. Next, the cells were harvested by centrifugation, washed three times with PBS (pH 7.2), fixed with 4% paraformaldehyde in phosphate buffer (pH 7.2), permeabilized with 0.1% Triton X-100 for 10 min and stained with 30 μg/mL propidium iodide for 15 min, in order to quantify the DNA content. Data acquisition and analysis were performed using a FACSCalibur flow cytometer (Becton-Dickinson, San Jose, CA, USA). A total of 10,000 events were acquired in the region that corresponds to the *T*. *foetus* population. Untreated parasites or cultures treated with 50 μM E-64d for 12 h were used as controls.

### Effects of lactacystin on the EFF formation

The parasites were treated with 10 μM or 20 μM lactacystin for 12 h, as mentioned above. Untreated parasites or cultures treated with 50 μM E-64d for 12 h were used as controls. Next, the parasites were harvested by centrifugation, washed three times with TYM medium without serum and resuspended in TYM medium supplemented with 10% foetal bovine serum in the absence of lactacystin or E-64d. The parasites were then submitted to the EFF induction assay as previously described. The EFF percentage was determined from counts of at least 1,000 parasites per sample after several time points (0, 0.5, 1, 2 and 3 h) using light microscopy.

### Scanning electron microscopy (SEM)

Parasites were washed with PBS and fixed in 2.5% glutaraldehyde in 0.1 M cacodylate buffer, pH 7.2. The cells were then post-fixed for 15 min in 1% OsO_4_, dehydrated in ethanol and critical point dried with liquid CO_2._ The dried cells were coated with gold-palladium to a thickness of 15 nm and then observed with a JEOL 5800 scanning electron microscope.

### Transmission electron microscopy (TEM)

#### Routine preparation

The parasites were washed with PBS and fixed in 2.5% glutaraldehyde in 0.1 M cacodylate buffer, pH 7.2. The cells were then post-fixed for 30 min in 1% OsO_4_, dehydrated in acetone and embedded in Epon (Polybed 812). Ultra-thin sections were harvested on 300 mesh copper grids, stained with 5% uranyl acetate and 1% lead citrate, and observed with a JEOL 1210 transmission electron microscope. The images were randomly acquired with a CCD camera system (MegaView G2, Olympus, Germany).

#### Negative staining

Two μL of cytosolic proteasome-enriched fraction (F3) were applied to glow-discharge carbon coated grid for 1 min and negatively stained with 1% uranyl acetate for 1 min. The grids were then dried and observed as described above.

### Statistical analysis

The results for all assays are the average of three independent experiments performed at least in duplicate. Statistical comparison was performed ANOVA test, using computer analysis (GraphPad Prism v. 5.00, California, USA). *P<*0.05 was considered to be statistically significant.

## Supporting Information

S1 Fig SEM of *T. foetus* obtained from a culture grown under standard conditions and at the end of the EFF induction assay. (A) Under standard conditions, the majority of the parasites exhibit a PS body with external flagella (arrows). (B) After the induction assay, the majority of the parasites are EFF. Bars, 10 μm.
(TIF)

S2 Fig Predicted full-length amino acid sequences of the representative members of the α-subunit gene family from *T. foetus* proteasome. The conserved domains identified by the NCBI CD-Search software are highlighted in yellow. The descriptions, NCBI identifiers, scores and KEGG orthology of the motifs are listed below each amino acid sequence.
(PDF)

S3 Fig Predicted full-length amino acid sequences of the representative members of the β-subunit gene family from *T. foetus* proteasome. The conserved domains identified by the NCBI CD-Search software are highlighted in yellow. The descriptions, NCBI identifiers, scores and KEGG orthology of the motifs are listed below each amino acid sequence.
(PDF)

S4 Fig Subcellular localisation of proteasome in dividing *T*. *foetus* using immunofluorescence. Parasites were incubated with the polyclonal anti-*T*. *cruzi* proteasome antibody followed by DAPI staining. Column 1, DIC microscopy; column 2, the labelling pattern obtained with anti-proteasome antibody; column 3, DAPI staining; column 4, merge. The labelling is found as punctate cytoplasmic structures and in the perinuclear region. First row: a PS parasite in a binary division stage. Note the presence of two nuclei. Second row: a pear-shaped parasite (arrow) can be seen in the process of budding from a multinucleated EFF. F, flagella. Bars, 4 μm.
(TIF)

S5 Fig Schematic of the preparation of 20S proteasome-enriched fraction from *T*. *foetus*. The parasites were disrupted with a Potter-type homogeniser and the cell homogenates were pre-cleared by centrifugation at 1,000 x g for 10 min. The supernatants (total cytosolic extract—TE) were collected and submitted to three sequential ultracentrifugation steps. The first step was at 12,000 x g for 20 min, the resulting supernatant fraction (F1) was then centrifuged at 150,000 x g for 1 h, and the supernatant from this (F2) was centrifuged at 150,000 x g for 6 h. The resulting pellet (F3), enriched in cytosolic proteasomes and the supernatant fraction (F4) were collected. All centrifugation procedures were performed at 4°C.
(TIF)

S6 Fig SEM of lactacystin-treated PS *T*. *foetus*. Parasites were incubated with 20 μM lactacystin for 12 h. (A) General view of parasite culture. (B) Detailed view of a parasite. Lactacystin did not induce alteration of external morphology of *T*. *foetus*. AF, anterior flagella; RF, recurrent flagellum, Ax, axostyle’s tip. Bars, A, 10 μm; B, 2 μm.
(TIF)

S7 Fig TEM of E-64d-treated PS *T*. *foetus*. Parasites were incubated with 50 μM E-64d for 12 h. Parasites exhibit alterations in lysosome-like structures (L) and more than one peripheral vesicle per hydrogenosomes (H). Other structures, such as endoplasmic reticulum (ER), nucleus (N) and axostyle (Ax) remain unaltered. Bars: A, 500 nm; B, 300 nm.
(TIF)

S8 Fig Effects of epoxomicin and bortezomib on growth and ultrastructure of PS *T*. *foetus*. (A-B) Growth curve of parasites treated with epoxomicin (A) and bortezomib (B). Parasites were initially cultured for 12 h at 37°C (initial inoculum: 1x10^4^ parasites/mL). After this period (arrow), 0.01, 0.1 and 1 μM of epoxomicin or 0.1, 0.5 and 1 μM of bortezomib were added to the culture medium and parasites were incubated for up to 30 h at 37°C. Cell growth was calculated after 6, 12, 18, 24 and 30 h of incubation. Parasites incubated with DMSO were used as control. Values are expressed as the means ± SD across three independent experiments, each performed in triplicate. (C-D) SEM (C) and TEM (D) of 0.01 μM epoxomicin-treated parasites for 6 h. (E-F) SEM (E) and TEM (F) of 0.1 μM bortezomib-treated parasites for 6 h. Similar effects were found with both compounds. The parasites exhibit several alterations indicative of cell death, such as appearance of wrinkled or rounded cells with externalised flagella (F), membrane blebbing (arrows), cell lysis (L), intense cytoplasmic (*) and nuclear (arrowhead) vacuolization, cytoplasmic disintegration (☆) and abnormal Golgi reduction (G). N, nucleus; C, costa; H, hydrogenosomes. Bars: A, 10 μm; B, D, 1 μm; C, 5 μm.
(TIF)

S1 Table UniProt accession number and the name of the proteasome sequences assigned for each species in the phylogenetic analyses. (DOCX)

S2 Table Amino acid sequence homology (% identity / similarity) of the *T*. *foetus*-20S proteasome α subunits using BLAST. (DOCX)

S3 Table Amino acid sequence homology (% identity / similarity) of the *T*. *foetus*-20S proteasome β subunits using BLAST. (DOCX)

S4 Table Summary of sequence comparisons of *T*. *foetus* -α proteasome subunits against their respective ortologues using BLAST. (DOCX)

S5 Table Summary of sequence comparisons of *T*. *foetus* -β proteasome subunits against their respective ortologues using BLAST. (DOCX)
