# Supplementary figures and images for: Characterisation of 20S Proteasome in Tritrichomonas foetus and Its Role during the Cell Cycle and Transformation into Endoflagellar Form
Source: PLoS One. 2015 Jun 5;10(6):e0129165. doi: 10.1371/journal.pone.0129165 (PMC4457923; doi:10.1371/journal.pone.0129165)

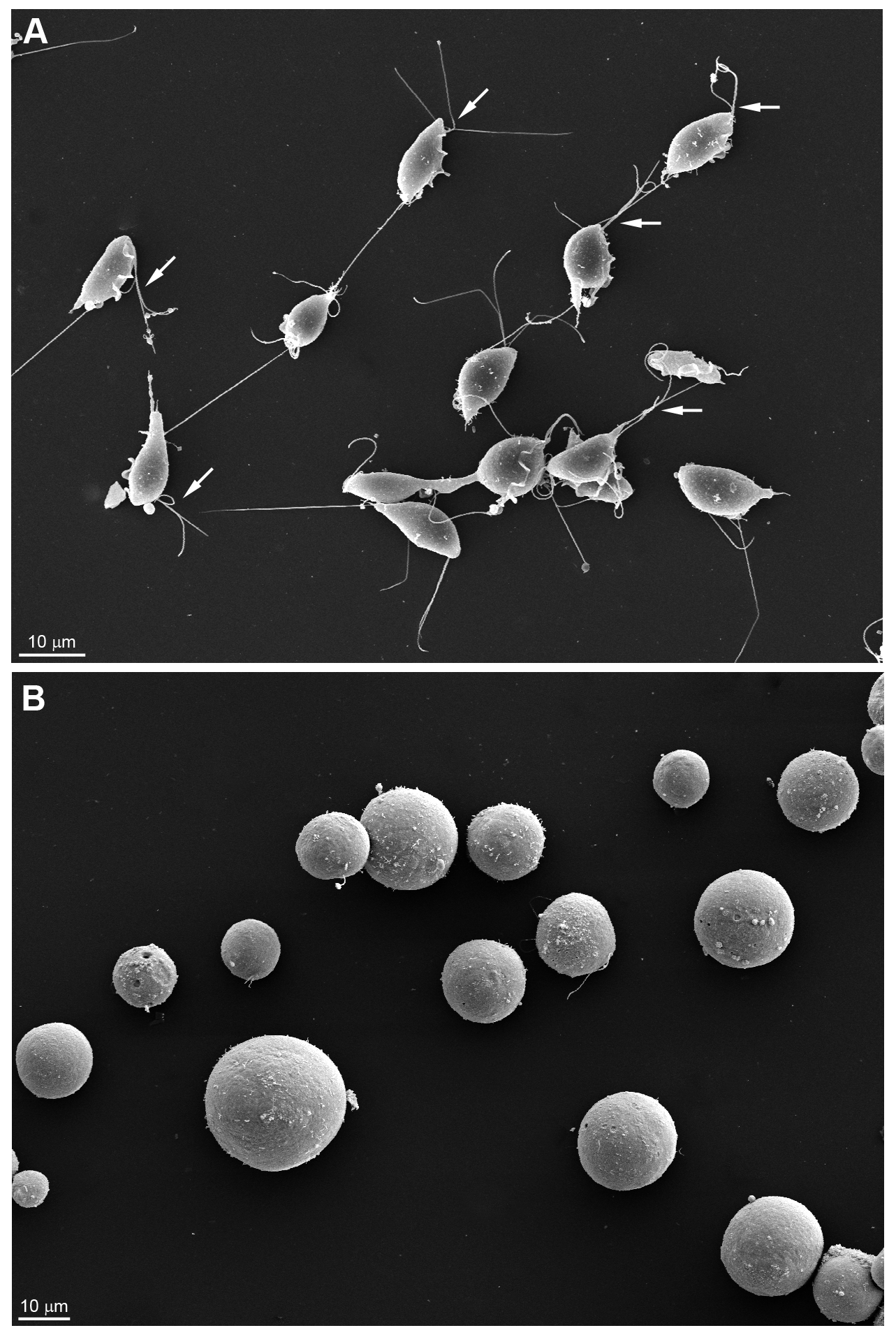

Supplement: S1 Fig — (A) Under standard conditions, the majority of the parasites exhibit a PS body with external flagella (arrows). (B) After the induction assay, the majority of the parasites are EFF. Bars, 10 μm. (TIF) [file pone.0129165.s001.tif]

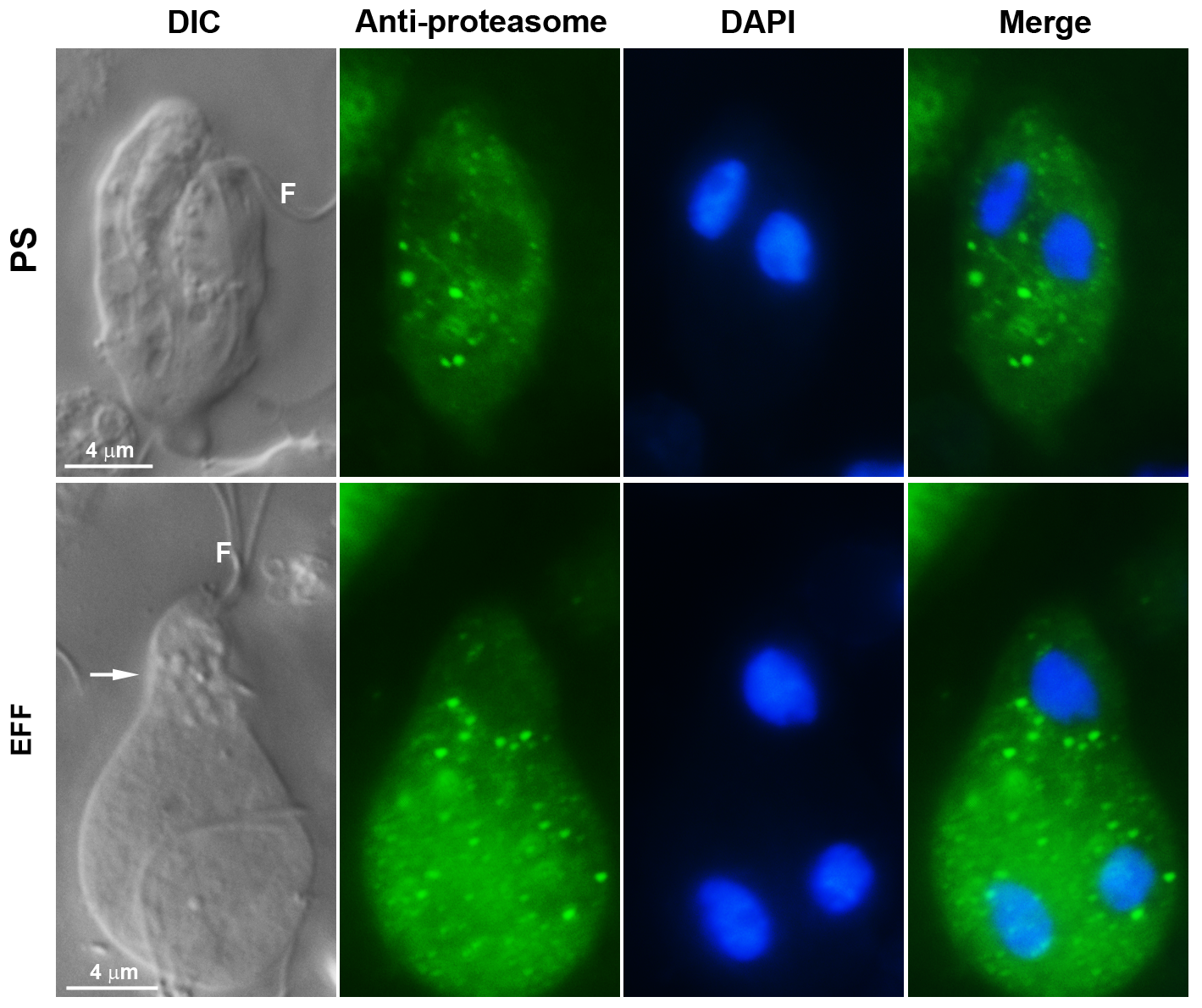

Supplement: S4 Fig — Parasites were incubated with the polyclonal anti-T. cruzi proteasome antibody followed by DAPI staining. Column 1, DIC microscopy; column 2, the labelling pattern obtained with anti-proteasome antibody; column 3, DAPI staining; column 4, merge. The labelling is found as punctate cytoplasmic structures and in the perinuclear region. First row: a PS parasite in a binary division stage. Note the presence of two nuclei. Second row: a pear-shaped parasite (arrow) can be seen in the process of budding from a multinucleated EFF. F, flagella. Bars, 4 μm. (TIF) [file pone.0129165.s004.tif]

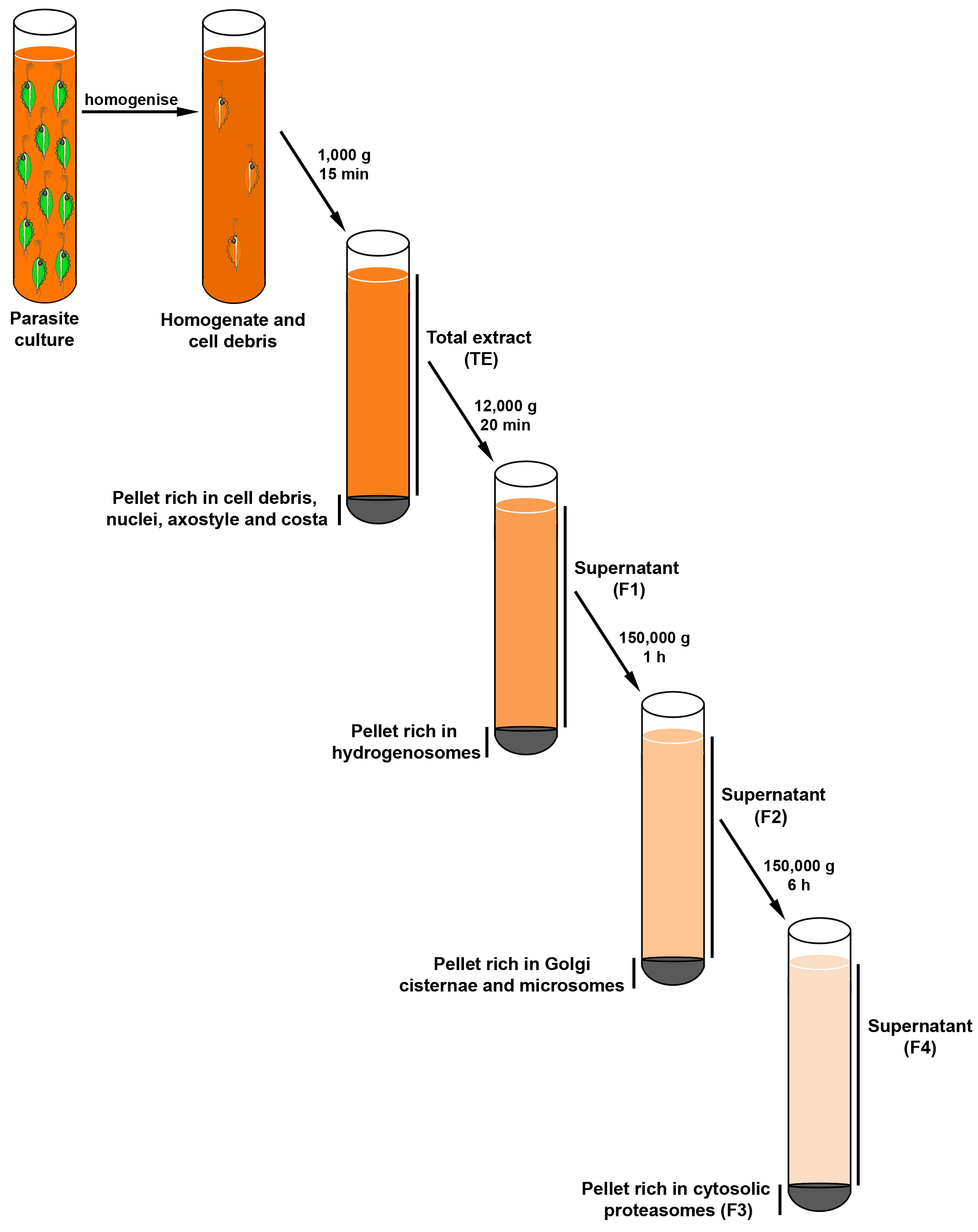

Supplement: S5 Fig — The parasites were disrupted with a Potter-type homogeniser and the cell homogenates were pre-cleared by centrifugation at 1,000 x g for 10 min. The supernatants (total cytosolic extract—TE) were collected and submitted to three sequential ultracentrifugation steps. The first step was at 12,000 x g for 20 min, the resulting supernatant fraction (F1) was then centrifuged at 150,000 x g for 1 h, and the supernatant from this (F2) was centrifuged at 150,000 x g for 6 h. The resulting pellet (F3), enriched in cytosolic proteasomes and the supernatant fraction (F4) were collected. All centrifugation procedures were performed at 4°C. (TIF) [file pone.0129165.s005.tif]

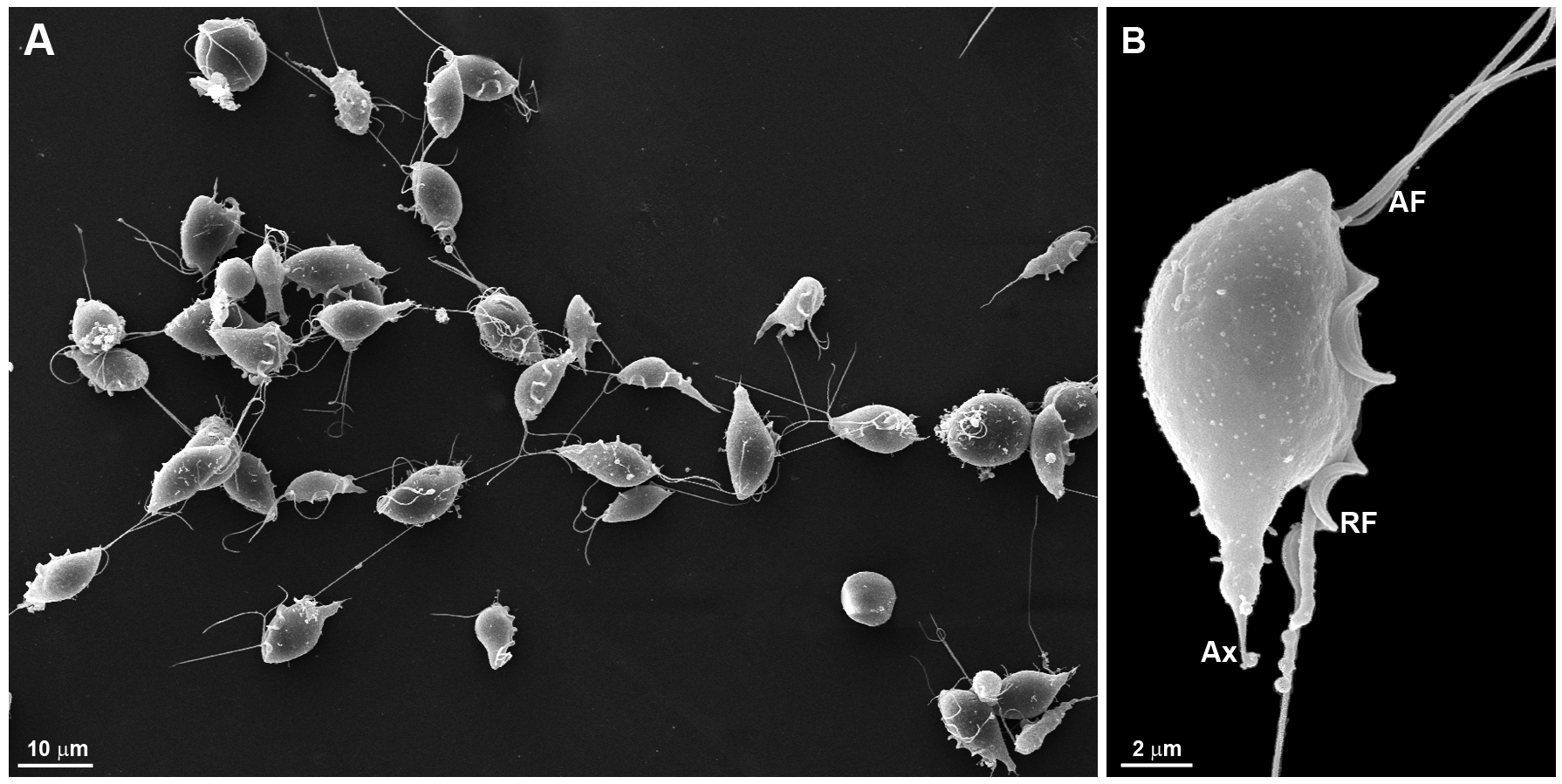

Supplement: S6 Fig — Parasites were incubated with 20 μM lactacystin for 12 h. (A) General view of parasite culture. (B) Detailed view of a parasite. Lactacystin did not induce alteration of external morphology of T. foetus. AF, anterior flagella; RF, recurrent flagellum, Ax, axostyle’s tip. Bars, A, 10 μm; B, 2 μm. (TIF) [file pone.0129165.s006.tif]

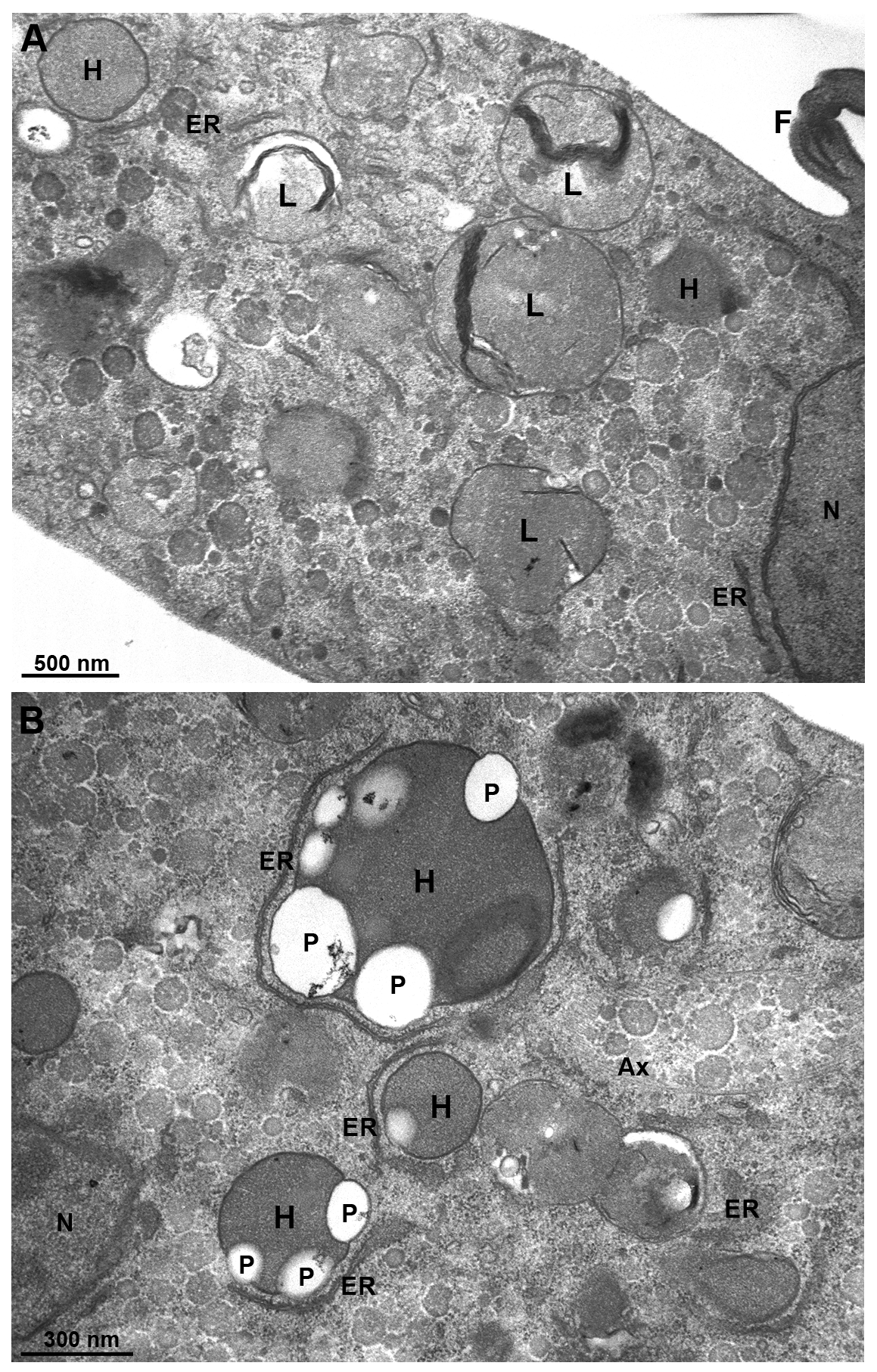

Supplement: S7 Fig — Parasites were incubated with 50 μM E-64d for 12 h. Parasites exhibit alterations in lysosome-like structures (L) and more than one peripheral vesicle per hydrogenosomes (H). Other structures, such as endoplasmic reticulum (ER), nucleus (N) and axostyle (Ax) remain unaltered. Bars: A, 500 nm; B, 300 nm. (TIF) [file pone.0129165.s007.tif]

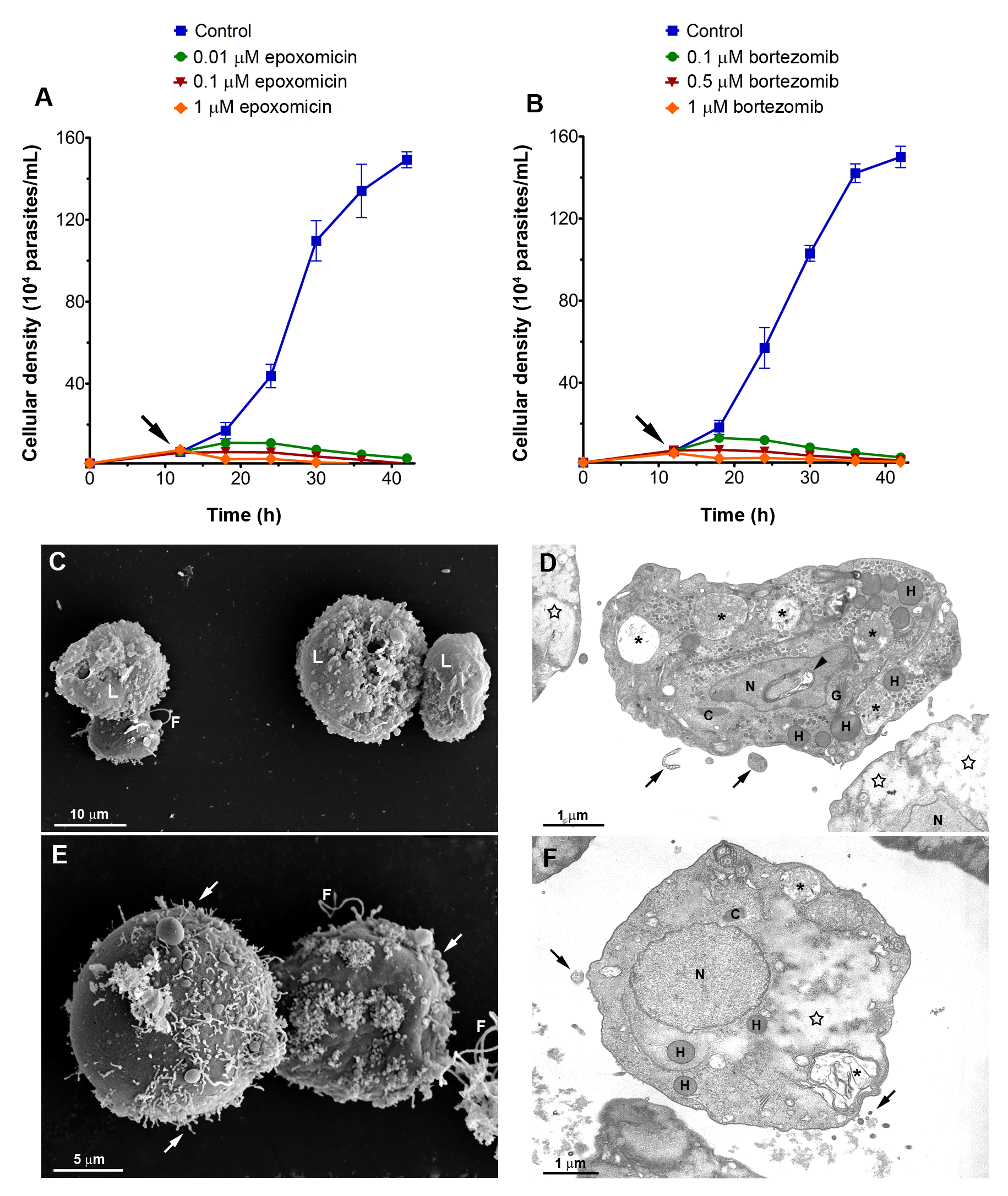

Supplement: S8 Fig — (A-B) Growth curve of parasites treated with epoxomicin (A) and bortezomib (B). Parasites were initially cultured for 12 h at 37°C (initial inoculum: 1x10^4^ parasites/mL). After this period (arrow), 0.01, 0.1 and 1 μM of epoxomicin or 0.1, 0.5 and 1 μM of bortezomib were added to the culture medium and parasites were incubated for up to 30 h at 37°C. Cell growth was calculated after 6, 12, 18, 24 and 30 h of incubation. Parasites incubated with DMSO were used as control. Values are expressed as the means ± SD across three independent experiments, each performed in triplicate. (C-D) SEM (C) and TEM (D) of 0.01 μM epoxomicin-treated parasites for 6 h. (E-F) SEM (E) and TEM (F) of 0.1 μM bortezomib-treated parasites for 6 h. Similar effects were found with both compounds. The parasites exhibit several alterations indicative of cell death, such as appearance of wrinkled or rounded cells with externalised flagella (F), membrane blebbing (arrows), cell lysis (L), intense cytoplasmic (*) and nuclear (arrowhead) vacuolization, cytoplasmic disintegration (☆) and abnormal Golgi reduction (G). N, nucleus; C, costa; H, hydrogenosomes. Bars: A, 10 μm; B, D, 1 μm; C, 5 μm. (TIF) [file pone.0129165.s008.tif]
