# Supplementary material for: Characterisation of 20S Proteasome in Tritrichomonas foetus and Its Role during the Cell Cycle and Transformation into Endoflagellar Form
Source: PLoS One. 2015 Jun 5;10(6):e0129165. doi: 10.1371/journal.pone.0129165 (PMC4457923; doi:10.1371/journal.pone.0129165)
Supplement: S2 Fig — The conserved domains identified by the NCBI CD-Search software are highlighted in yellow. The descriptions, NCBI identifiers, scores and KEGG orthology of the motifs are listed below each amino acid sequence. (PDF) [file pone.0129165.s002.pdf]

| | Sequence | Motif | Identifier | Score | E-value | KEGG orthology |
|---|---|---|---|---|---|---|
| **TfoetusA1** | MSSSADRFITIFSQEGRLWQVEYAFKAVKQAEVTAIGAKGANSLVVAVQKKVQDKLIDPSTVTHMFRIT<br>EHVGACLIGLLPDVLYICRRLRYDAAQFEFKNGFEIPVNILSSRLSEIHQIESQYSGARPTGVSAIIFG<br>FEPSKNDFALYKVEPSGFTSGYRAVSCGVKEVEAMSALEKKYESFPTEKETAEFVISTLQTVVGLDFEA<br>REVEVAVVTRENTAYRVMKADEVEKVLTAVAEKD | Proteasome_alpha_type_6 | cd03754 | 264.10 | 8.83E-89 | 20S proteasome subunit alpha 1 (K02730) |
| **TfoetusA2** | MGDSDFSLTTFSSGGKLGQIEHALKAVSLSGQCVGVKAKDGAVIACESKPPSPLAERDTNLKVQAINPN<br>VGCVYSGIITDYRVLLKKLRKEAMKYKLRLGVEMPTREVVKSAAAINQSYTQSGGVRPFGVSLLIIGWE<br>DIPTLWQVDPSGTFWAWKATALGKRSDGSRTFLERRYNEDLSVDDAIHTAISTLKEGFDGQLTKDLIEI<br>GVVDKTHKFRTLSTEEIEDFLTEV | Proteasome_alpha_type_2 | cd03750 | 324.66 | 1.85E-112 | 20S proteasome subunit alpha 2 (K02726) |
| **TfoetusA3** | MSYRYDAGTTTFSSDGRILQVEYAIQSINQAGTAIGVQFSNGVVLAAEKKNTGRLVDYLFPEKMAKLDE<br>HVITALAGMTADANNLVDFMRSLAQSYLKTYGEPMPVEQLVRRVCDKKHSYTQYGGLRPYGVSFLIAGY<br>DRHKGCQLYLTDPSGNFGGWKATAIGENNQTAQSILKSSYKDDMNATEAMDLTVKVLCKTLDSTSLSAD<br>KLEFSVLQYSEKTGPKVRILTTAEVEHLMKRFEDTIKATTEEKE | Proteasome_alpha_type_4 | cd03752 | 328.92 | 5.75E-114 | 20S proteasome subunit alpha 3 (K02728) |
| **TfoetusA4** | MSHYARSITRFSPDGRLFQIDHAQAAVQRGTTVVAVQSKDLIVIAVEKLTIAKLQDPCTFRKVVA<br>LDDHVMCAFAGLHADARVLVQKAQVECQSHRLTYEDPITVERIARHIATLQLKYTQSGGVRPFGV<br>ATLVCGFDSTTKQPHIYETLPSGAYAEWKARTIGRHDQTVMEYLEKHYKDDMTDDDAIKMAVGSL<br>LEVVENGAKNLEVALMKCGEPMVLMKEEQLAPLIEAINKK | Proteasome_alpha_type_7 | cd03755 | 322.39 | 8.68E-112 | 20S proteasome subunit alpha 4 (K02731) |
| **TfoetusA5** | MFQSSSEYDRNVNTFSPDGRLLQVEYAIEAVKLGSSAIAILCPEGVVFAVEKRLSSPLLIPSSVERVYA<br>IDEHIGVVLAGYPADGRTMVDHMRVAAQDHRFSFGEPIGIRAVTESVCDLALSFGEGRRKREGQMSRPF<br>GAALLVSGIDNGKPFLFHTDPSGTYTQCRARAIGGGSEGAETILRDSYHDGMTLEEAENLALSTLRQVI<br>QEKLSENNIEVACANVQTGRFKVYSAEQRQAIVERLPPPLIQE | Proteasome_alpha_type_5 | cd03753 | 331.22 | 5.56E-115 | 20S proteasome subunit alpha 5 (K02729) |
| **TfoetusA6** | MFRSRYDGDTTTFSPEGRLLQVENAMKAVQQGMSTVGIRSQTHAVIACIMHSPSEFSSFQPKIFKIDEH<br>IGVTISGLTADGRGLCKMLRSECLRHKFVYGTESKVSTLADFIADRSQNKTQKVGKRPYGVGLLMIGAN<br>PADGPRLFETCPSGQNYEYDAQSIGRRSQAAKTYLEQNLPEFHNSTRDELIKHALKALFDCRAKEENGL<br>ECFAVGVVGVDEPFMIIEGDALRPYFSE | Proteasome_alpha_type_1 | cd03749 | 297.28 | 6.74E-102 | 20S proteasome subunit alpha 6 (K02725) |
| **TfoetusA7** | MSGAGSGYDFNAFTFSPDGRLFQVEYATKAVDKEALALGVRCSDGVLFAVEKNLSSKLLTPGGNPRTFW<br>IDTHIACATVGYRPDSYAAVLQARKEAANYFETFGCKITVPELVSRVAHTFHSSHAFSSIRPYGCALLI<br>GSLEGPSLYALEPNGQYFGYYACCFGKGSSLARAELQRTDWGEKTVEEAVPLVANIIKELHEAQNKTWE<br>IEMLWVCQASEGKPQKVPESLFVAAQ | Proteasome_alpha_type_3 | cd03751 | 247.19 | 3.13E-82 | 20S proteasome subunit alpha 7 (K02727) |
