# Supplementary material for: Characterisation of 20S Proteasome in Tritrichomonas foetus and Its Role during the Cell Cycle and Transformation into Endoflagellar Form
Source: PLoS One. 2015 Jun 5;10(6):e0129165. doi: 10.1371/journal.pone.0129165 (PMC4457923; doi:10.1371/journal.pone.0129165)
Supplement: S3 Fig — The conserved domains identified by the NCBI CD-Search software are highlighted in yellow. The descriptions, NCBI identifiers, scores and KEGG orthology of the motifs are listed below each amino acid sequence. (PDF) [file pone.0129165.s003.pdf]

TfoetusB1

Sequence

MTNPNALVEMTGTTLFAIKTADGVVFAADSRSTAGSTVACRTSNKITQISSKVFVARTGSTADTQALAR
YGHYILNVLTMNSESEVKRPVLVASQYLRKVLQSNKEYLSASLICGGIEDDGTPSLYEINTSGMAIPRD
FCLNGSGSSYILAFCDEKYRDNMTMEEATKFAIEAVTNAIIRDGASGGFVNIVQVTKDQTKRFVVMPKD
QPYNHSVVKT

Motif	Identifier	Score	E-value	KEGG orthology
Proteasome_beta_type_6	cd03762	197.06	2.65E-63	20S proteasome subunit beta 1 (K02738)

TfoetusB2

Sequence

MQNKGFDFDLYARNESLKVQPKKLKTGTTIAAAVFDGGVVLGADTRATAGPVVAVKDEFKIHYISDNIW
CCGAGTAADNDQITTLISSKLRLYTMNSGMQPRVYQAATLLTSRLFKYQGYIEAYLIVAGVDFQGPSVY
TMYANGSCSKSPFSTMGSGSMAAVSVLEAGWKPKMSEEACKELVADAIEAGITNDLGSGSNVNLCVIKN
TGHEYISHYRVTNERTFRMNAPIKRGTIEVISSTTKPLHPPEVHLEILDGVAEEAAA

Motif	Identifier	Score	E-value	KEGG orthology
Proteasome_beta_type_7	cd03763	292.18	8.63E-100	20S proteasome subunit beta 2 (K02739)

TfoetusB3

Sequence

MSGGIDTQNGVSVVAMAGDHCVAIAADRRLGAQMQTVSTNFKKIFQINDYIHVGMSGLATDIDTVYEKL
RYDVNLLELREERQLEPIRFMSLVRSLLYEHRFGPYFVSPVIAGLDPATNEPYLATSDSIGAFNQPKDF
AVAGTSEESLYGICESMWRPGLNPDELFDVISQCLIAAVERDGLSGWGAVVHIITPESVVVKEIKTRMD

Motif	Identifier	Score	E-value	KEGG orthology
Proteasome_beta_type_3	cd03759	317.65	1.23E-110	20S proteasome subunit beta 3 (K02735)

TfoetusB4

Sequence

MLSIVGLCGPDWVLIAADSSVSSSIICMSEEYDRIAEIGKHNALALAGETGDALQLSEYIIGNVALYKF
INSVELTTDAISHYIRNEMAKAVRKNPYQVNMLLAGYDEKPSLYYLDYLGTRQKIPFGAQGYCAYFVLS
VFDKFYEENMSLEKGKEVMKKALDQIKQRFTIAPHGFIVKLIDANGIQKINLD

Motif	Identifier	Score	E-value	KEGG orthology
Proteasome_beta_type_2	cd03758	236.33	5.68E-79	20S proteasome subunit beta 4 (K02734)

TfoetusB5

Sequence

MSGLQFPQHDVIDGENQAPLQPANVEDPCGFIKNHLHFNYTNDAPDSNRIAAYHGTTCLSFIYKGGIVV
AVDSRATGGSFIFSGTVMKILDIAPNMIGTMAGGAADCQYWLRSLSRICRLHNFRYQQPLTVAAASKIL
VNELYSYKGYNLSIGTMICGYDSTGPHIFYIDNDGSRLEGKRFSVGSGSTYAYGVLDTCYKYEMTKEEA
CDLGRKAIYHATYRDSGSGGRVTVVHIDENGAQRISQTDVFDIHDFDKKVE

Motif	Identifier	Score	E-value	KEGG orthology
Proteasome_beta_type_5	cd03761	313.80	2.04E-108	20S proteasome subunit beta 5 (K02737)

TfoetusB6

Sequence

MHSEVAGKIEDKKGGWSPYDNHGGTSVGIVGKDFVAIGTDTRLSSNYSISCRHKSRVFQMTSKAMIVAT
GFDGDIDAFVTRIRQILVRYQQEHFKEMSTESLALCVSNILYSKRFFPYYINILVGGIGLKDEGLLYGY
DPVGTLECLNYDAHGTGSPMAMPILDNHFGSMHHNTTPFPHPEVDDAVNLIRDIMASVSERDIYTGDCL
QVAVMKSDGNLTITEYELPAH

Motif	Identifier	Score	E-value	KEGG orthology
Proteasome_beta_type_1	cd03757	214.04	2.28E-69	20S proteasome subunit beta 6 (K02732)

TfoetusB7

Sequence

MSGYKKSVQNPITTTNSIIAAKYKDGILLASDRAVSYGSCFKFANVSHFAKLTPNIIIGGTGELADFQE
LVDVLRSIIVDEECKNNGESLTPSEVSNYIKRLMYERRSKMNPFVMRCILAGIDKDGSKLLTATDLYGT
QWEDEYVASGYGAHMQGVQIPKALKSADVTRDVVMDAIKEVFIGLTARHSTMSGPIEFVDVTANGIEFL
DPIEIVPNWDVLDEDWAQ

Motif	Identifier	Score	E-value	KEGG orthology
Ntn_hydrolase super family	cl00467	173.53	7.65E-54	20S proteasome subunit beta 7 (K02736)
