# Supplementary material for: Characterisation of 20S Proteasome in Tritrichomonas foetus and Its Role during the Cell Cycle and Transformation into Endoflagellar Form
Source: PLoS One. 2015 Jun 5;10(6):e0129165. doi: 10.1371/journal.pone.0129165 (PMC4457923; doi:10.1371/journal.pone.0129165)
Supplement: S1 Table — (DOCX) [file pone.0129165.s009.docx]

**Table S1**. UniProt accession number and the name of the proteasome sequences assigned for each species in the phylogenetic analyses.

| **Species** | **α-type subunits** | | **β-type subunits** | |
| --- | --- | --- | --- | --- |
|  | **UniProt accession nº** | **Name** | **UniProt accession nº** | **Name** |
| *Trichomonas vaginalis* | A2F568 | TvagA1 | A2E7Z2 | TvagB1 |
|  | A2FJV7 | TvagA2 | A2F2T6 | TvagB2 |
|  | A2FT79 | TvagA3 | A2F3H9 | TvagB3 |
|  | A2E5C0 | TvagA4 | A2F8W4 | TvagB4 |
|  | A2FCM7 | TvagA5 | A2DD57 | TvagB5 |
|  | A2E1I9 | TvagA6 | A2F716 | TvagB6 |
|  | A2D8G5 | TvagA7 | A2F3X4 | TvagB7 |
| *Trypanosoma cruzi* | Q4E4G8 | TcruA1 | Q4DW25 | TcruB1 |
|  | Q4CKY8 | TcruA2 | Q4E4R6 | TcruB2 |
|  | Q4DIK4 | TcruA3 | Q4DHA9 | TcruB3 |
|  | Q4CYK5 | TcruA4 | Q4CU77 | TcruB4 |
|  | Q4DAW0 | TcruA5 | Q4D8U7 | TcruB5 |
|  | Q4D5X3 | TcruA6 | Q4DND1 | TcruB6 |
|  | Q4CTU6 | TcruA7 | Q4D579 | TcruB7 |
| *Dictyostelium discoideum* | Q54XM7 | DdisA1 | Q55GJ6 | DdisB1 |
|  | Q54DM7 | DdisA2 | Q54QR2 | DdisB2 |
|  | P34119 | DsisA3 | Q55D66 | DdisB3 |
|  | P34120 | DdisA4 | Q55DY7 | DdisB4 |
|  | Q55G04 | DdisA5 | Q54BC8 | DdisB5 |
|  | Q27562 | DdisA6 | Q86A21 | DdisB6 |
|  | Q27563 | DdisA7 | Q556Q0 | DdisB7 |
| *Saccharomyces cerevisiae* | P21243 | ScerA1 | P38624 | ScerB1 |
|  | P23639 | ScerA2 | P25043 | ScerB2 |
|  | P23638 | ScerA3 | P25451 | ScerB3 |
|  | P40303 | ScerA4 | P22141 | ScerB4 |
|  | P32379 | ScerA5 | P30656 | ScerB5 |
|  | P40302 | ScerA6 | P23724 | ScerB6 |
|  | P21242 | ScerA7 | P30657 | ScerB7 |
| *Arabidopsis thaliana* | O81147 | AthaA1 | Q8LD27 | AthaB1 |
|  | O23708 | AthaA2 | O23710 | AthaB2 |
|  | O81148 | AthaA3 | Q9XI05 | AthaB3 |
|  | P30186 | AthaA4 | O23714 | AthaB4 |
|  | O81149 | AthaA5 | O23717 | AthaB5 |
|  | O23712 | AthaA6 | P42742 | AthaB6 |
|  | O23715 | AthaA7 | Q7DLR9 | AthaB7 |
| *Caenorhabditis elegans* | O17586 | CeleA1 | Q966I8 | CeleB1 |
|  | Q27488 | CeleA2 | O62102 | CeleB2 |
|  | Q9N599 | CeleA3 | Q23237 | CeleB3 |
|  | Q95005 | CeleA4 | P91477 | CeleB4 |
|  | Q95008 | CeleA5 | Q9XUV0 | CeleB5 |
|  | O44156 | CeleA6 | P34286 | CeleB6 |
|  | Q09583 | CeleA7 | P90868 | CeleB7 |
| *Drosophila melanogaster* | Q9XZJ4 | DmelA1 | A0AQH0 | DmelB1 |
|  | P40301 | DmelA2 | Q8T915 | DmelB2 |
|  | Q9VA12 | DmelA3 | Q9XYN7 | DmelB3 |
|  | Q24178 | DmelA4 | Q8T8U1 | DmelB4 |
|  | Q95083 | DmelA5 | Q7K148 | DmelB5 |
|  | P12881 | DmelA6 | P40304 | DmelB6 |
|  | Q9V5C6 | DmelA7 | Q9VNA5 | DmelB7 |
| *Homo sapiens* | P60900 | HsapA1 | P28072 | HsapB1 |
|  | P25787 | HsapA2 | Q99436 | HsapB2 |
|  | P25789 | HsapA3 | P49720 | HsapB3 |
|  | Q8TAA3 | HsapA4 | P49721 | HsapB4 |
|  | P28066 | HsapA5 | P28074 | HsapB5 |
|  | P25786 | HsapA6 | P20618 | HsapB6 |
|  | P25788 | HsapA7 | P28070 | HsapB7 |
