# Supplementary material for: Characterisation of 20S Proteasome in Tritrichomonas foetus and Its Role during the Cell Cycle and Transformation into Endoflagellar Form
Source: PLoS One. 2015 Jun 5;10(6):e0129165. doi: 10.1371/journal.pone.0129165 (PMC4457923; doi:10.1371/journal.pone.0129165)
Supplement: S2 Table — (DOCX) [file pone.0129165.s010.docx]

Table S2. Amino acid sequence homology (% identity / similarity) of the *T. foetus*-20S proteasome α subunits using BLAST.

| **α subunits** | TfoetusA2 | TfoetusA3 | TfoetusA4 | TfoetusA5 | TfoetusA6 | TfoetusA7 |
| --- | --- | --- | --- | --- | --- | --- |
| TfoetusA1 | 27 / 50 | 30 / 48 | 25 / 50 | 28 / 46 | 31 / 48 | 29 / 47 |
| TfoetusA2 | ---- | 32 / 54 | 36 / 56 | 30 / 56 | 32 / 51 | 30 / 49 |
| TfoetusA3 | ---- | ---- | 36 / 57 | 36 / 55 | 32 / 51 | 30 / 50 |
| TfoetusA4 | ---- | ---- | ---- | 34 / 55 | 32 / 50 | 27 / 47 |
| TfoetusA5 | ---- | ---- | ---- | ---- | 34 / 53 | 36 / 50 |
| TfoetusA6 | ---- | ---- | ---- | ---- | ---- | 29 / 44 |
