# Supplementary material for: Characterisation of 20S Proteasome in Tritrichomonas foetus and Its Role during the Cell Cycle and Transformation into Endoflagellar Form
Source: PLoS One. 2015 Jun 5;10(6):e0129165. doi: 10.1371/journal.pone.0129165 (PMC4457923; doi:10.1371/journal.pone.0129165)
Supplement: S3 Table — (DOCX) [file pone.0129165.s011.docx]

Table S3. Amino acid sequence homology (% identity / similarity) of the *T. foetus*-20S proteasome β subunits using BLAST.

| **α subunits** | TfoetusB2 | TfoetusB3 | TfoetusB4 | TfoetusB5 | TfoetusB6 | TfoetusB7 |
| --- | --- | --- | --- | --- | --- | --- |
| TfoetusB1 | 30 / 46 | 24 / 42 | 25 / 45 | 31 / 46 | 23 / 40 | 22 / 43 |
| TfoetusB2 | ---- | 15 / 28 | 21 / 46 | 30 / 47 | 14 / 30 | 13 / 28 |
| TfoetusB3 | ---- | ---- | 20 / 44 | 23 / 40 | 22 / 42 | 20 / 35 |
| TfoetusB4 | ---- | ---- | ---- | 24 / 42 | 23 / 45 | 26 / 46 |
| TfoetusB5 | ---- | ---- | ---- | ---- | 17 / 39 | 23 / 40 |
| TfoetusB6 | ---- | ---- | ---- | ---- | ---- | 24 / 40 |
