# Supplementary material for: Characterisation of 20S Proteasome in Tritrichomonas foetus and Its Role during the Cell Cycle and Transformation into Endoflagellar Form
Source: PLoS One. 2015 Jun 5;10(6):e0129165. doi: 10.1371/journal.pone.0129165 (PMC4457923; doi:10.1371/journal.pone.0129165)
Supplement: S4 Table — (DOCX) [file pone.0129165.s012.docx]

| ***T. foetus* paralogous^a^** | **Ortologous group^b^** | **% identity / similarity** | **E-value** | **Cover (%)** | **Score** |
| --- | --- | --- | --- | --- | --- |
| TfoetusA1 | TvagA1 | 63 / 80 | 8.00E-116 | 100 | 323 |
|  | TcruA1 | 34 / 61 | 5.00E-54 | 97 | 165 |
|  | DdisA1 | 38 / 66 | 1.00E-64 | 95 | 192 |
|  | ScerA1 | 35 / 59 | 1.00E-49 | 97 | 154 |
|  | AthaA1 | 44 / 67 | 7.00E-61 | 85 | 181 |
|  | CeleA1 | 39 / 65 | 4.00E-65 | 99 | 193 |
|  | DmelA1 | 41 / 66 | 3.00E-70 | 99 | 207 |
|  | HsapA1 | 43 / 66 | 5.00E-71 | 99 | 209 |
| TfoetusA2 | TvagA2 | 84 / 88 | 2.00E-142 | 100 | 390 |
|  | TcruA2 | 47 / 68 | 2.00E-58 | 77 | 173 |
|  | DdisA2 | 50 / 71 | 1.00E-78 | 97 | 227 |
|  | ScerA2 | 49 / 64 | 3.00E-72 | 96 | 212 |
|  | AthaA2 | 54 / 71 | 9.00E-87 | 100 | 248 |
|  | CeleA2 | 50 / 67 | 1.00E-75 | 99 | 233 |
|  | DmelA2 | 53 / 70 | 1.00E-82 | 97 | 238 |
|  | HsapA2 | 53 / 70 | 9.00E-82 | 98 | 235 |
| TfoetusA3 | TvagA3 | 87 / 94 | 2.00E-170 | 100 | 462 |
|  | TcruA3 | 50 / 68 | 3.00E-75 | 85 | 221 |
|  | DdisA3 | 54 / 72 | 1.00E-93 | 94 | 267 |
|  | ScerA3 | 53 / 73 | 2.00E-81 | 85 | 236 |
|  | AthaA3 | 58 / 75 | 4.00E-91 | 84 | 261 |
|  | CeleA3 | 50 / 70 | 2.00E-78 | 94 | 228 |
|  | DmelA3 | 42 / 61 | 8.00E-62 | 94 | 186 |
|  | HsapA3 | 50 / 70 | 1.00E-82 | 94 | 239 |
| TfoetusA4 | TvagA4 | 56 / 74 | 5.00E-97 | 98 | 275 |
|  | TcruA4 | 49 / 72 | 6.00E-81 | 93 | 234 |
|  | DdisA4 | 52 / 76 | 2.00E-91 | 98 | 261 |
|  | ScerA4 | 48 / 71 | 7.00E-83 | 98 | 239 |
|  | AthaA4 | 58 / 78 | 2.00E-67 | 64 | 196 |
|  | CeleA4 | 53 / 73 | 1.00E-79 | 90 | 231 |
|  | DmelA4 | 43 / 66 | 8.00E-72 | 99 | 211 |
|  | HsapA4 | 53 / 74 | 8.00E-89 | 95 | 254 |
| TfoetusA5 | TvagA5 | 81 / 91 | 1.00E-156 | 100 | 427 |
|  | TcruA5 | 51 / 72 | 2.00E-92 | 97 | 264 |
|  | DdisA5 | 52 / 73 | 2.00E-98 | 97 | 279 |
|  | ScerA5 | 51 / 68 | 7.00E-90 | 97 | 258 |
|  | AthaA5 | 53 / 76 | 6.00E-98 | 97 | 278 |
|  | CeleA5 | 50 / 70 | 6.00E-82 | 88 | 237 |
|  | DmelA5 | 53 / 73 | 1.00E-84 | 88 | 244 |
|  | HsapA5 | 46 / 67 | 5.00E-59 | 73 | 176 |
| TfoetusA6 | TvagA6 | 73 / 85 | 9.00E-130 | 99 | 358 |
|  | TcruA6 | 41 / 66 | 4.00E-68 | 99 | 202 |
|  | DdisA6 | 45 / 66 | 5.00E-76 | 99 | 221 |
|  | ScerA6 | 44 / 60 | 3.00E-67 | 99 | 198 |
|  | AthaA6 | 49 / 68 | 4.00E-77 | 97 | 225 |
|  | CeleA6 | 45 / 66 | 1.00E-68 | 95 | 203 |
|  | DmelA6 | 44 / 65 | 1.00E-69 | 95 | 206 |
|  | HsapA6 | 49 / 70 | 4.00E-40 | 47 | 124 |
| TfoetusA7 | TvagA7 | 62 / 77 | 4.00E-114 | 98 | 318 |
|  | TcruA7 | 37 / 56 | 6.00E-53 | 96 | 161 |
|  | DdisA7 | 39 / 57 | 1.00E-61 | 100 | 184 |
|  | ScerA7 | 40 / 58 | 4.00E-53 | 93 | 164 |
|  | AthaA7 | 40 / 60 | 2.00E-66 | 100 | 197 |
|  | CeleA7 | 35 / 52 | 1.00E-46 | 100 | 145 |
|  | DmelA7 | 40 / 59 | 2.00E-55 | 93 | 169 |
|  | HsapA7 | 41 / 61 | 2.00E-66 | 97 | 197 |

Table S4. Summary of sequence comparisons of *T. foetus* –α proteasome subunits against their respective ortologues using BLAST

^a^ See Table 1 for accession numbers

^b^ See Table S1 for accession number
