# Supplementary material for: Characterisation of 20S Proteasome in Tritrichomonas foetus and Its Role during the Cell Cycle and Transformation into Endoflagellar Form
Source: PLoS One. 2015 Jun 5;10(6):e0129165. doi: 10.1371/journal.pone.0129165 (PMC4457923; doi:10.1371/journal.pone.0129165)
Supplement: S5 Table — (DOCX) [file pone.0129165.s013.docx]

| ***T. foetus* paralogous^a^** | **Ortologous group^b^** | **% identity / similarity** | **E-value** | **Cover (%)** | **Score** |
| --- | --- | --- | --- | --- | --- |
| TfoetusB1 | TvagB1 | 52 / 67 | 4.00E-74 | 95 | 214 |
|  | TcruB1 | 35 / 55 | 2.00E-38 | 91 | 124 |
|  | DdisB1 | 35 / 57 | 1.00E-41 | 91 | 130 |
|  | ScerB1 | 34 / 56 | 2.00E-40 | 90 | 127 |
|  | AthaB1 | 39 / 57 | 3.00E-42 | 87 | 133 |
|  | CeleB1 | 37 / 57 | 1.00E-40 | 92 | 129 |
|  | DmelB1 | 41 / 59 | 3.00E-41 | 90 | 130 |
|  | HsapB1 | 38 / 55 | 2.00E-33 | 75 | 109 |
| TfoetusB2 | TvagB2 | 62 / 73 | 2.00E-117 | 95 | 329 |
|  | TcruB2 | 50 / 66 | 2.00E-73 | 83 | 218 |
|  | DdisB2 | 52 / 68 | 1.00E-81 | 95 | 238 |
|  | ScerB2 | 46 / 65 | 6.00E-73 | 89 | 215 |
|  | AthaB2 | 51 / 68 | 4.00E-76 | 83 | 223 |
|  | CeleB2 | 45 / 65 | 3.00E-60 | 76 | 183 |
|  | DmelB2 | 36 / 57 | 8.00E-50 | 91 | 157 |
|  | HsapB2 | 49 / 64 | 3.00E-82 | 95 | 239 |
| TfoetusB3 | TvagB3 | 71 / 85 | 2.00E-113 | 98 | 314 |
|  | TcruB3 | 48 / 67 | 2.00E-69 | 98 | 202 |
|  | DdisB3 | 48 / 69 | 1.00E-74 | 96 | 215 |
|  | ScerB3 | 51 / 69 | 9.00E-75 | 96 | 215 |
|  | AthaB3 | 52 / 74 | 4.00E-79 | 96 | 226 |
|  | CeleB3 | 47 / 67 | 1.00E-74 | 99 | 215 |
|  | DmelB3 | 53 / 70 | 9.00E-83 | 96 | 236 |
|  | HsapB3 | 51 / 72 | 7.00E-82 | 98 | 233 |
| TfoetusB4 | TvagB4 | 74 / 89 | 9.00E-111 | 100 | 306 |
|  | TcruB4 | 34 / 56 | 4.00E-38 | 93 | 120 |
|  | DdisB4 | 42 / 65 | 5.00E-51 | 91 | 153 |
|  | ScerB4 | 36 / 64 | 3.00E-51 | 97 | 154 |
|  | AthaB4 | 42 / 65 | 2.00E-56 | 97 | 167 |
|  | CeleB4 | 30 / 55 | 7.00E-30 | 98 | 98.6 |
|  | DmelB4 | 31 / 57 | 1.00E-37 | 98 | 119 |
|  | HsapB4 | 40 / 63 | 2.00E-40 | 83 | 125 |
| TfoetusB5 | TvagB5 | 76 / 86 | 1.00E-114 | 96 | 398 |
|  | TcruB5 | 55 / 72 | 3.00E-76 | 88 | 225 |
|  | DdisB5 | 52 / 74 | 5.00E-89 | 89 | 256 |
|  | ScerB5 | 49 / 68 | 2.00E-75 | 90 | 223 |
|  | AthaB5 | 51 / 68 | 2.00E-78 | 84 | 230 |
|  | CeleB5 | 49 / 63 | 5.00E-71 | 85 | 211 |
|  | DmelB5 | 56 / 75 | 6.00E-80 | 76 | 234 |
|  | HsapB5 | 52 / 74 | 3.00E-82 | 80 | 239 |
| TfoetusB6 | TvagB6 | 63 / 78 | 2.00E-105 | 96 | 295 |
|  | TcruB6 | 31 / 54 | 3.00E-40 | 92 | 128 |
|  | DdisB6 | 36 / 57 | 2.00E-48 | 92 | 149 |
|  | ScerB6 | 35 / 57 | 1.00E-44 | 94 | 140 |
|  | AthaB6 | 40 / 60 | 5.00E-57 | 89 | 171 |
|  | CeleB6 | 30 / 54 | 2.00E-38 | 93 | 124 |
|  | DmelB6 | 35 / 60 | 6.00E-48 | 88 | 148 |
|  | HsapB6 | 37 / 60 | 1.00E-50 | 88 | 155 |
| TfoetusB7 | TvagB7 | 50 / 69 | 9.00E-80 | 95 | 229 |
|  | TcruB7 | 32 / 53 | 2.00E-36 | 91 | 117 |
|  | DdisB7 | 30 / 52 | 3.00E-37 | 93 | 120 |
|  | ScerB7 | 32 / 55 | 4.00E-33 | 82 | 110 |
|  | AthaB7 | 31 / 55 | 6.00E-40 | 96 | 127 |
|  | CeleB7 | 27 / 50 | 3.00E-24 | 94 | 84.7 |
|  | DmelB7 | 28 / 51 | 2.00E-33 | 88 | 110 |
|  | HsapB7 | 31 / 53 | 2.00E-40 | 95 | 129 |

Table S5. Summary of sequence comparisons of *T. foetus* –β proteasome subunits against their respective ortologues using BLAST

^a^ See Table 1 for accession numbers

^b^ See Table S1 for accession numbers
